# The Microbiome of the Seaweed Cultivar *Ulva compressa* (Chlorophyta) and Its Persistence Under Micropollutant Exposure

**DOI:** 10.1111/1758-2229.70230

**Published:** 2025-11-10

**Authors:** Justus Hardegen, Gabriel Amend, Thomas Wichard

**Affiliations:** ^1^ Friedrich Schiller University Jena Institute for Inorganic and Analytical Chemistry Jena Germany; ^2^ Jena School for Microbial Communication Jena Germany

**Keywords:** algae, algal growth and morphogenesis‐promoting factors, antibiotics, core microbiome, pesticides, pollutants, seaweed

## Abstract

The green macroalga *Ulva* demonstrates exceptional growth rates, robustness, adaptability and potential for nitrogen and phosphorus removal; thus, it is a promising candidate for wastewater treatment and bioremediation. However, micropollutants in wastewater pose a potential threat to the holobiont. We explored the effects of emerging contaminants found in groundwater and wastewater on the microbiome of the cultivar *Ulva compressa* (conspecific with *Ulva mutabilis* ). We identified the core microbiome by comparing the microbiome of the long‐term cultivar (cultivated under laboratory conditions for over 70 years) with the native microbiome of *U. compressa* . Long‐term cultivation was found to homogenise and reduce microbiome diversity; however, key functional taxa, including algal growth and morphogenesis‐promoting bacteria, persisted. We subsequently challenged the core microbiome of the *U. compressa* cultivar with four antibiotics (chloramphenicol, erythromycin, oxytetracycline and sulfamethoxazole), two herbicides (atrazine, glyphosate) and three endocrine disruptors (bisphenol A, estradiol and ethinylestradiol). The micropollutants exerted distinct impacts, with antibiotics showing stronger effects than hormonal disruptors, which *Ulva* rapidly removes from the culture medium. In contrast, the microbiome did not contribute to the removal of these substances. These results indicate that although *Ulva*'s microbiome is sensitive to environmental change, key functions with positive implications for aquaculture and ecosystem management are retained.

## Graphical abstract

Despite reduced bacterial diversity due to cultivation and subsequent micropollutant treatment, key bacterial functions are retained, promoting macroalgal growth and morphogenesis.

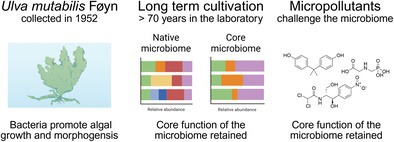

## 1 Introduction

Human activities increasingly contribute to global environmental pollution, particularly in regions lacking strict environmental regulations (Gavrilescu et al. 2015). Thus, pollutants such as eutrophying nutrients, heavy metals and micropollutants (MPs) pose a threat to water quality and ecosystems. Conventional treatment technologies are often ineffective, costly or infeasible for these pollutants, necessitating alternative approaches.

Algae‐based phytoremediation is a promising, sustainable and cost‐effective strategy for wastewater treatment (Mohsenpour et al. 2021; Viegas et al. 2021; El Semary 2023; Rahhou et al. 2023). Among algae, macroalgae such as *Ulva* (sea lettuce) are particularly attractive because of their rapid growth, adaptability and ease of harvesting (Qiu et al. 2017; Liu et al. 2020). *Ulva* species can efficiently remove nitrogen, phosphorus and heavy metals from polluted water bodies (Qiu et al. 2017; Liu et al. 2020).

In addition to its physiological traits, *Ulva* maintains a complex symbiotic relationship with its microbiome, forming a functional holobiont (Spoerner et al. 2012; Ghaderiardakani et al. 2022). The holobiont comprises the host and associated microorganisms, including bacteria, archaea and viruses, whose interactions support host health, development and environmental resilience (Zilber‐Rosenberg and Rosenberg 2008). Specific bacterial strains provide *Ulva* with algal growth and morphogenesis‐promoting factors (AGMPFs), which are critical for its development and ecological function (Ghaderiardakani et al. 2019; Wichard 2023). For example, bacteria of the genera *Maribacter* or *Zobellia* release thallusin to induce cell differentiation (i.e., the phenocopy of *Maribacter* sp. MS6 defines functional clade I), and *Rhodobacteriaceae* (i.e., the phenocopy of *Roseovarius* sp. MS2 defines functional clade II) can promote *Ulva* growth (Wichard 2023). Both functional clades are essential, and their combination is critical for the complete growth and morphogenesis of *Ulva*. In turn, *Ulva* secretes chemoattractants and carbon compounds that shape its microbial community (Kessler et al. 2018; Califano et al. 2020).

This mutualistic microbiome implies a stable core component of bacterial taxa that persist across environmental conditions and are essential for host function. These core microbes produce vital compounds for nutrient uptake, morphogenesis, vitamin synthesis and pathogen resistance. In contrast, a dysbiotic microbiome may impair these functions, compromising the health and remediation ability of *Ulva*. While research has characterised the microbiomes of environmentally friendly and aquaculture‐grown *Ulva* (Burke et al. 2011; Roth‐Schulze et al. 2018; van der Loos et al. 2023, 2024), the effects of MP exposure on this core microbiome remain poorly understood.

Defining a core microbiome comprising the microbial taxa or functions consistently associated with *Ulva* across diverse conditions is fundamental to understanding host–microbe interactions. This process must strike a balance between experimental control and ecological relevance. Few long‐term macroalgae are available; thus, comparing the long‐term cultured strain of *Ulva compressa* (cultivar *Ulva mutabilis* [Føyn]), FSU‐UM1‐41, with collected samples from the natural environment presents a unique opportunity to identify the core microbiome. Here, we hypothesised that key core microbiome members are those ASVs consistently present in both long‐term cultivars and environmental samples.

*Ulva mutabilis* (Føyn) was collected in 1952 from Ria Formosa (Portugal) and has been cultured under laboratory conditions for more than 70 years (Føyn 1958; Løvlie 1964; Stratmann et al. 1996; Wichard 2015). The aforementioned approach leverages the stability of microbial communities maintained in controlled conditions over time, which can help isolate persistent, host‐associated microorganisms from those acquired opportunistically from the environment or transient strains.

Moreover, we aimed to examine how exposure to emerging compounds influences the composition and stability of *Ulva*'s core microbiome during extended laboratory cultivation. We investigated whether these MPs disrupt beneficial microbial communities, reduce the production of AGMPFs, and negatively affect *Ulva* growth and bioremediation capacity. Conversely, specific microbial taxa may increase the resilience of *Ulva* (Hmani et al. 2024) or directly participate in pollutant degradation. Importantly, we used cultivars with a stable microbiome, avoiding variability caused by shifting abiotic variables, seasonal fluctuations, stochastic microbial colonisation processes and transient settlement on the surface (Wahl et al. 2012; Saha et al. 2024) that complicate cross‐habitat comparisons.

To explore microbiome dynamics, we selected nine representative MPs on the basis of their frequent detection in wastewater, surface water and groundwater, representing various substance classes (Kummerer 2009; Luo et al. 2014; Das et al. 2017; Sousa et al. 2018; Tran et al. 2018; Nguyen et al. 2022). These include:

1. Antibiotics: Broad‐spectrum agents such as chloramphenicol (CAP), erythromycin (ERY), oxytetracycline (OTC) and sulfamethoxazole (SMX) are commonly used in aquaculture and are known to inhibit bacterial protein or folic acid synthesis (Steinhilber et al. 2010; Chen et al. 2020; Höck and Ziesing 2020).
2. Herbicides: Atrazine (ATZ) and glyphosate (PMG), both of which target photosynthetic or amino acid synthesis pathways present in *Ulva* and associated bacteria (Rose et al. 2016; Montiel‐Leon et al. 2019; Barnett and Gibson 2020).
3. Endocrine disruptors: Bisphenol A (BPA), estradiol (E_2_) and ethinylestradiol (EE_2_) are frequently detected in effluents and are known for their subtle yet significant ecological effects (Allard 2014; Laurenson et al. 2014; Michalowicz 2014; Hardegen et al. 2021).

Understanding how these substances affect the cultivar microbiome of *U. compressa* will provide critical insights into the resilience and functionality of algal holobionts in polluted environments. This knowledge is essential for optimising the application of *Ulva* in sustainable water treatment and aquaculture systems.

## 2 Materials and Methods

### 2.1 *Ulva* Cultivation

*Ulva compressa* , an established model organism (Blomme et al. 2023; Wichard 2023) that has been cultivated for 70 years, was selected to decipher the core microbiome of *Ulva*: haploid gametophytes of *Ulva mutabilis* Føyn (morphotype ‘wild type’; locus typicus: Ria Formosa, Portugal, strain FSU‐UM1‐41) have been propagated with their native microbiome since 1952 (Føyn 1958). These organisms were cultivated for 40 years in filtered seawater and then in artificial seawater (UCM: *Ulva* culture medium). The strain is identical to the genome‐sequenced strains *U. mutabilis* (FSU‐UM1‐41, CCAP 6038/2) and is conspecific with *Ulva compressa* (De Clerck et al. 2018; Steinhagen et al. 2019). The cultivar is referred to as *U. compressa* throughout this publication. In addition, *U. compressa* (rfu‐61) was sampled from Ria Formosa (Faro, Portugal) for comparison with the cultivar holobiont (Alsufyani et al. 2014; Grueneberg et al. 2016). *Ulva* was cultured in artificial seawater (UCM) at 18°C ± 2°C with a light/dark cycle of 17/7 h and a light intensity of 40–80 μmol photons m^−2^ s^−1^ (Califano and Wichard 2018; Nahor et al. 2021).

### 2.2 Evaluating the Changes in the Microbial Community in Response to Chemical Stressors

The wild‐type holobiont (approximately 200 mg of fresh weight) was incubated for 14 days with antibiotics, herbicides or hormones in 10 mL of UCM, with four replicates per compound (Table S1). Analytical standards of ATZ, ERY, 17α‐EE_2_, 17β‐E_2_, OTC, PMG and SMX were purchased from Sigma Aldrich/Merck (Germany), BPA from Alfa Aesar (USA) and CAP from Fluka/Honeywell (USA).

As a criterion, the concentrations were chosen to maximise the effect on the microbiome while avoiding damage to the algae, corresponding to 10% (EC_10_) toxicity towards *Ulva* as previously reported by Hardegen et al. (2023) (the EC_10_ value is the concentration of a substance that causes a 10% effect compared to a control in a specified biological or ecological test).

As negative controls, five replicates were sampled before and five after incubation without additional chemicals. Since all the compounds except PMG were dissolved in methanol before addition, all the treatments with more than 0.1% *v*/*v* of methanol were supplemented with methanol for a total of 2.1% *v*/*v* (Table S1). Methanol was chosen over the more commonly used DMSO because the latter displays negative chemotaxis towards symbiotic bacteria (Kessler et al. 2018). Four replicates with 2.1% methanol (*v*/*v*) were also prepared as a control.

### 2.3 Determination of the Removal of MPs From the Culture Medium

The removal of MPs from the culture medium by *Ulva* or abiotic processes was determined via liquid chromatography coupled with mass spectrometry, per established procedures (Hardegen et al. 2023, 2025). The 14‐day removal efficiency of the laboratory *Ulva* culture with its associated microbiome (*Ulva* strain FSU‐UM1‐41) was compared to that of the abiotic controls under the same light regime and to that of the tripartite community of *Ulva compressa–Roseovarius* sp. MS2–Maribacter sp. MS6 (*Ulva* strain FSU‐UM5‐1; morphotype ‘slender’). The experiment was performed in quadruplicate in small culture flasks with a 10 mL volume.

### 2.4 DNA Extraction

DNA was extracted from 200 mg (fresh weight) *Ulva* thallus (Califano et al. 2020) using DNeasy PowerSoil Pro Kits (Qiagen, Germany), following the manufacturer's instructions under strictly sterile conditions at a laminar flow bench.

### 2.5 Illumina Libraries and Sequencing

Nextera was used to barcode two‐step PCR libraries (Illumina, USA) via the locus‐specific primer pair 515F_5N (5′‐GTGYCAGCMGCCGCGGTAA‐3′) (Parada et al. 2016) and 806R (5′‐GGACTACNVGGGTWTCTAAT‐3′) (Apprill et al. 2015) for sequencing of the V4 region of the bacterial 16S rRNA gene: the first step involved 20 PCR cycles, and the second involved 10. The PCR libraries were subsequently sequenced on an Illumina MiSeq platform using a v2 500 cycles kit (Microsynth AG, Balgach, Switzerland). Sequence data are available at the National Centre for Biotechnology Information, project number PRJNA828511, including the BioSample accession numbers SAMN48728450–SAMN48728504 (Table S2).

### 2.6 Amplicon Metagenomics Data Analysis

Analysis was performed for two groups: (1) laboratory culture samples in combination with environmental samples and (2) laboratory culture samples alone (Table S3). The produced paired‐end reads that passed the chastity filter (Illumina) were subjected to demultiplexing and trimming of Illumina adaptor residuals via bcl2fastq software (version v2.20.0.422). The quality of the reads was checked with FastQC software (version 0.11.9). Sequencing reads that fell below an average Phred score of 25 or contained any uncalled bases (N) were removed from further analyses. The locus‐specific V4 primers were trimmed from the sequencing reads with Cutadapt v3.2 software (Martin 2011). Paired‐end reads were discarded if the primer could not be trimmed. The trimmed forward and reverse reads of each paired‐end read were merged *in silico* to reconstruct the sequenced molecule, considering a minimum overlap of 15 bases via USEARCH version 11.0.667 (Edgar 2010). Merged reads containing ambiguous bases or outliers regarding the expected amplicon size distribution were also removed.

The remaining reads were denoised via the UNOISE algorithm (Edgar 2016a) implemented in USEARCH to form amplicon sequence variants (ASVs). Singletons and chimaeras were discarded. The resulting ASV abundance table was then filtered for possible barcode contamination via the UNCROSS algorithm (Edgar 2018). ASV sequences were compared to the reference sequences of the RDP 16S database (Cole et al. 2014), and taxonomies were predicted via the SINTAX algorithm (Edgar 2016b) implemented in USEARCH.

Alpha diversity was estimated via the Richness (observed), Simpson and Shannon indices. Rarefaction analysis was conducted to estimate the coverage of the captured metagenome in contrast to the potential metagenome.

Beta diversity was calculated via the weighted UniFrac distance method according to rarefied ASV abundance counts per sample. Community composition was compared using PERMANOVA based on Bray–Curtis dissimilarities (1000 permutations).

Alpha and beta diversity calculations and rarefaction analysis were performed with the R software packages phyloseq v1.26.1 (McMurdie and Holmes 2013) and vegan v2.5‐5 (Oksanen 2022). To detect differentially abundant ASVs depending on the collected sample metadata, differential ASV analysis on normalised abundance counts was performed with the R software package DESeq2 v1.26.0 (Love et al. 2014).

### 2.7 Data Visualisation

The data were visualised with OriginPro 2024 (OriginLab, USA). For the abundance plots, reads with taxonomical assignments were used. ASVs with significant differences in the number of reads (*p* < 0.01, fold change > 4) between the control after 14 days and the methanol control were identified and plotted separately. A detrended correspondence analysis (DCA) was performed for all samples and the laboratory samples alone. In this process, reads were normalised by sum to obtain relative counts (per object). Pearson correlation of samples was calculated from reads without normalisation. Moreover, the UniFrac distance matrix provided further insights into the phylogenetic dissimilarity between microbial communities exposed to various chemical treatments and control conditions. Each value in the matrix represents the degree of difference between two microbial communities, with lower values indicating greater similarity and higher values reflecting more substantial differences. We identified patterns of similarity and divergence among the samples by visually inspecting the matrix. Clustered heatmaps were created for individual samples and treatments. In this process, reads were normalised by sum to receive relative counts (per object) and autoscaled (mean‐centred and divided by the standard deviation of each variable). The columns and rows were both clustered via the Ward method with the Euclidean distance.

### 2.8 Further Statistical Analysis

The control and methanol control groups were compared after 14 days, and ASVs exhibiting a significant difference (*p* < 0.01) and a fold change > 4 were identified. The absolute abundance of all ASVs in the culture samples (controls and treatments) was compared via ANOVA with the Dunn–Šidák post hoc test.

## 3 Results

Two experimental approaches were followed. First, the core microbiome of *U. compressa* was determined by comparing long‐term cultured *U. compressa* (cultivar FSU‐UM1‐41) to collected *U. compressa* (environmental sample RFU‐61). Second, the effects of nine MPs on the microbiome of the cultivar were assessed.

Rarefaction analysis based on observed ASVs, Shannon and Simpson indices confirmed that sequencing depth was sufficient across all samples, as most curves reached or approached a plateau (Figure S1). PERMANOVA revealed that the condition (environmental sample versus long‐term cultivar) alone explained ~30% of the variance in microbial community composition (*R* ^2^ = 0.27, *F* = 2.94, *p* = 0.021). Moreover, the combined factor of MP‐treatment × time (MP exposure across two time points) explained ~80% of the variance (*R* ^2^ = 0.82, *F* = 15.5, *p* < 0.001), supporting the strong and interactive effects of MP treatments and temporal dynamics on microbiome structure.

### 3.1 The *Ulva* Cultivar Presented Reduced Microbiome Diversity Under Laboratory Conditions

**FIGURE 1:**
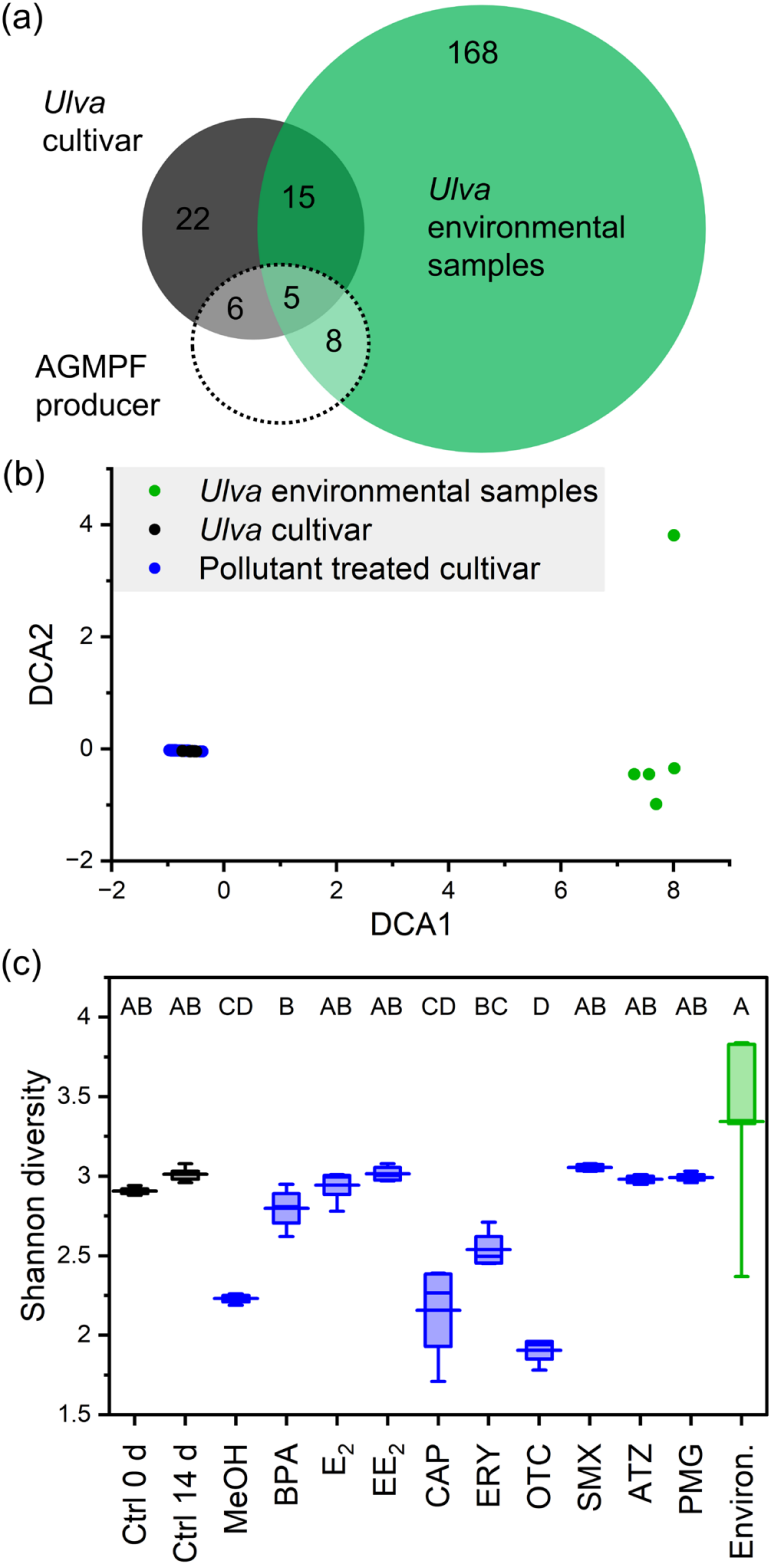
Identification of the core microbiome of *Ulva*. (a) The microbiome diversity, expressed as the number of ASVs, of a laboratory culture of *U. compressa* , which has been maintained for more than 70 years, was compared with that of environmental samples collected in Ria Formosa (Faro, Portugal). ASVs indicating the production of alga growth and morphogenesis‐promoting factors (AGMPFs) were identified, and chloroplast ASVs were excluded. Five essential AGMPF producers were present in both environmental samples and cultivar. (b) Detrended correspondence analysis and (c) Shannon diversity of environmental samples, the cultivar and the treated cultivar. The box is drawn from the first to the third quartile and separated by the median line; the whiskers represent the 10th–90th percentile, and the long horizontal line represents the mean (*n* = 4–5). ATZ, atrazine; BPA, bisphenol A; CAP, chloramphenicol; Ctrl d 0, control before incubation; Ctrl d 14, control after 14 days of incubation; E_2_, estradiol; EE_2_, ethinylestradiol; ERY, erythromycin; MeOH, methanol control; OTC, oxytetracycline; PMG, glyphosate; SMX, sulfamethoxazole.

Comparison of the cultivar *U. compressa* with native strains revealed reduced diversity in long‐term cultures, retaining only a few key taxa essential for *Ulva* growth and morphogenesis. 48 ASVs characterised the cultivar. In contrast, the native surface microbiome of *U. compressa* was characterised by approximately 196 ASVs (Figure 1a). Beta diversity across all samples was assessed via a DCA plot, which was used to compare the native microbiome of *Ulva compressa* with that of the long‐term cultivar and with that of samples exposed to MP treatments (Figure 1b). The separation between the cultivar and environmental samples in the DCA plot indicated substantial differences in their microbiomes. However, the changes in the cultivar microbiome induced by the MP treatments were relatively minor compared with the inherent variability observed among the natural samples from Ria Formosa (Portugal).

The Shannon diversity index (*H*′) supported these observations, suggesting greater diversity in the microbiome of an environmental sample of *Ulva* (*H*′ > 3.5) than in that of the laboratory‐cultured samples (*H*′ < 3.0), independent of antibiotic treatment. However, treatments such as CAP and OTC significantly decreased diversity (*H*′ < 2.5) (Figure 1c). The effect of MeOH on diversity is also considerable, as MeOH functions as an alternative carbon source rather than the photosynthates provided by *U. compressa* . Cultivars presented a stable but minimal core microbiome, which was further reduced by some of the treatments, such as CAP and OTC. Those H′ are typical for environmental microbiome samples, whereas the Simpson index (*D*) ranked around 0, indicating a high diversity without dominating species (Table S3).

### 3.2 Strains Retained During Long‐Term Cultivation

The comparison of the microbiome in the long‐term cultivar with that in an environmentally collected sample of *U. compressa* revealed 20 shared ASVs (Figure 1a), covering 13 families, with Rhodobacteraceae (*n* = 4) and Flavobacteriaceae (*n* = 4) as the most dominant (Table 1). Seven shared ASVs belonging to genera that can provide *Ulva* with essential AGMPFs (Grueneberg et al. 2016; Ghaderiardakani et al. 2017; Weiss et al. 2017) were distributed across functional clades I (ASV #10, #21, #23, #41) and II (#20, #77, #84), phenocopying *Maribacter* sp. MS6 and *Roseovarius* sp. MS2, respectively. The assignment of two species to functional clade II was conducted using unpublished data. While the abundance and presence of shared ASVs varied between the samples, three ASVs (#10, #12 and #22) were characterised by a high number of reads in most of the samples, and three (#20, #53 and #84) were characterised by their presence in most of the samples.

**TABLE 1:** Abundance of core species.

ASV ID	Taxonomy			Abundance in samples					
	Species	FC	Conf.	Cultivar (*n* = 10)		Treatments (*n* = 40)		Environment (*n* = 5)	
				Mean reads	Occurence	Mean reads	Occurence	Mean reads	Occurence
#26	*Amaricoccus kaplicensis*		2%	397	100%	432	100%	35	20%
#167	*Anoxybacillus contaminans*		3%	3	90%	3	53%	37	100%
#173	*Bacillus fumarioli*		3%	4	80%	3	55%	39	100%
#22	*Blastopirellula cremea*		44%	374	100%	567	100%	245	80%
#171	*Enterococcus cecorum*		81%	3	100%	4	60%	32	100%
#144	*Filomicrobium fusiforme*		62%	3	90%	8	90%	3	20%
#53	*Hyphomonas beringensis*		18%	80	100%	54	100%	9	100%
#77	*Jannaschia faecimaris*	ii ^a^	42%	1	50%	2	13%	240	100%
#21	*Maribacter antarcticus*	I	65%	609	100%	522	100%	22	20%
#10	*Maribacter chungangensis*	I	63%	2318	100%	2006	98%	768	100%
#41	*Maribacter chungangensis*	I	23%	103	100%	104	100%	6	20%
#50	*Marinobacter* sp.		100%	48	100%	74	100%	3	40%
#20	*Marivita* sp.	II^a^	22%	448	100%	734	100%	38	80%
#206	*Microcella alkaliphila*		32%	4	90%	4	85%	3	40%
#23	*Muricauda aquimarina*	I	24%	398	100%	558	100%	25	20%
#28	*Pseudohaliea rubra*		21%	221	100%	292	98%	16	20%
#12	*Rhodovulum mangrovi*		10%	521	100%	1867	100%	159	80%
#221	*Sphingorhabdus flavimaris*		85%	4	60%	4	85%	5	40%
#84	*Sulfitobacter dubius*	II	26%	8	100%	22	98%	16	80%
#181	*Tepidiphilus margaritifer*		81%	4	80%	3	63%	23	100%

*Note:* Species were found in the cultivar, environmental samples and treatments. The table corresponds to the Venn diagram's shared circle of cultivar and environmental sample (*n* = 20). The functional clade (FC) indicates the morphogenetic activity of the bacteria. FC I: Induction of rhizoid and cell wall formation. FC II: Induction of growth during germination. Confidence (Conf.) of species assignment. Abundance is expressed as the mean number of reads per sample and the percentage of occurrence.

^a^ Assignment to functional clade is based on unpublished data (J. Ulrich and T. Wichard).

### 3.3 Removal of MPs During Cultivation

**FIGURE 2:**
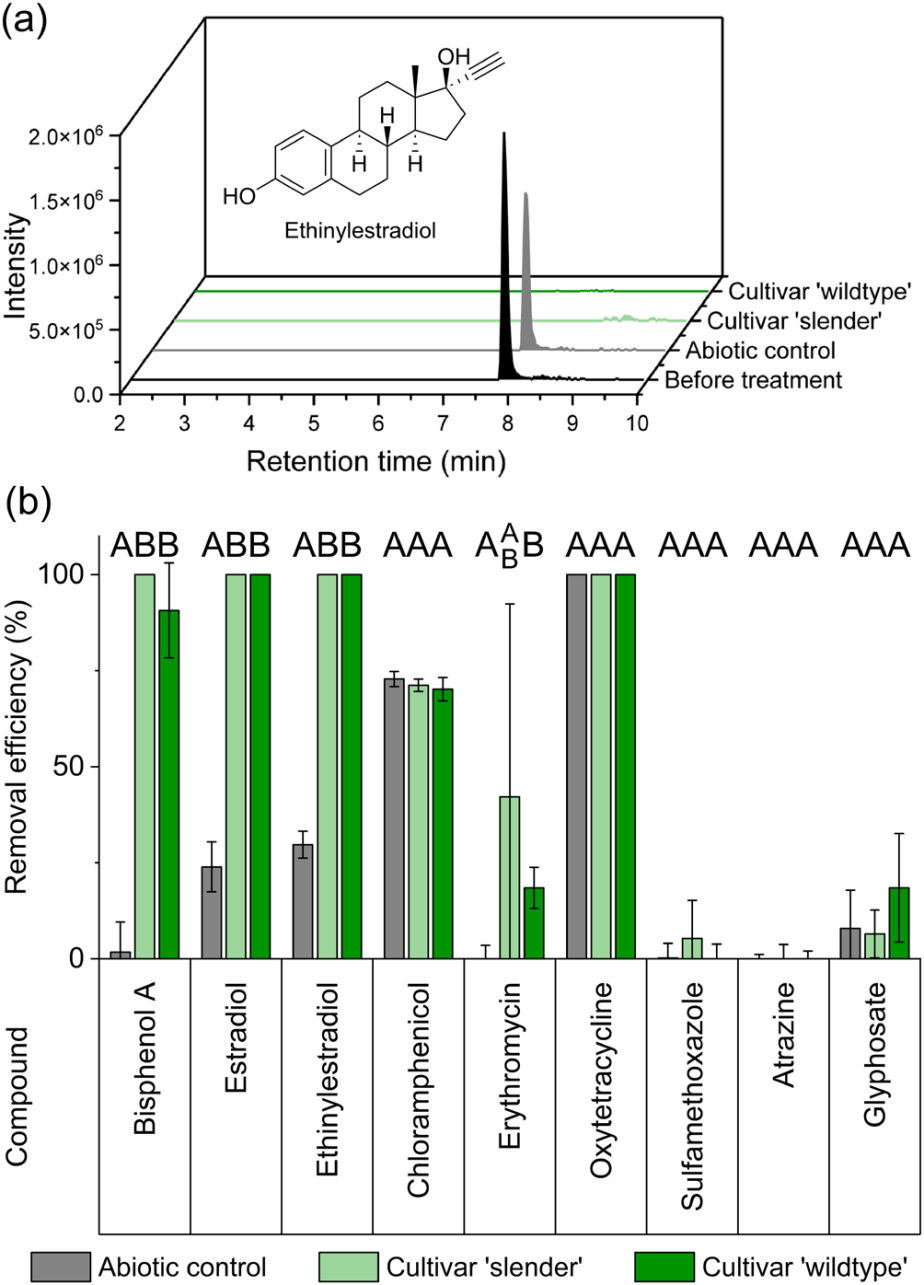
Removal efficiency of selected micropollutants over 14 days. (a) Example of the removal of ethinylestradiol: Reduction in peak areas in the extracted ion chromatograms of different treatments. (b) Comparison of removal in sterile‐filtered supernatant (grey, *n* = 3), tripartite *Ulva* (light green, strain FSU‐UM5‐1, *n* = 3), and wild‐type *Ulva* (dark green, strain FSU‐UM1‐41, *n* = 4) cultures. Wild‐type *Ulva* refers to a laboratory culture that retains a core microbiome (48 ASVs) after long‐term incubation. Efficiency is presented as the mean, with error bars indicating the standard deviation. The removal efficiencies of the different conditions were compared for each compound using an ANOVA with a Dunn‐Sidak post hoc test; columns that do not share a letter were significantly different (*p* < 0.05).

The MP removal efficiency of *Ulva* and its cultivated microbiome was compared to that of the filtered supernatant and the designed tripartite community *Ulva‐Roseovarius‐Maribacter* (including the two essential bacteria). ATZ and SMX persisted in the medium under all conditions. In addition, the removal efficiency varied depending on the specific treatment and MP. Importantly, no significant differences were detected between treatments where *Ulva* was associated either with its native microbiome or with the synthetic tripartite community (Figure 2). *Ulva* was the most efficient at removing any endocrine disruptor, such as bisphenol A, estradiol and ethinylestradiol; these compounds were almost entirely removed except under abiotic conditions.

In addition, glyphosate, CAP and OTC were removed to varying extents (11% for glyphosate, 71% for CAP and 100% for OTC), although their removal rates did not differ significantly across the evaluated conditions, suggesting a strong abiotic influence under the experimental setup. In contrast, ERY remained fully stable under abiotic conditions but was partially removed in the presence of *Ulva*, indicating a potential biotic contribution to its degradation (Figure 2).

### 3.4 MPs and MeOH Influenced the Microbiome Composition of *Ulva*

**FIGURE 3:**
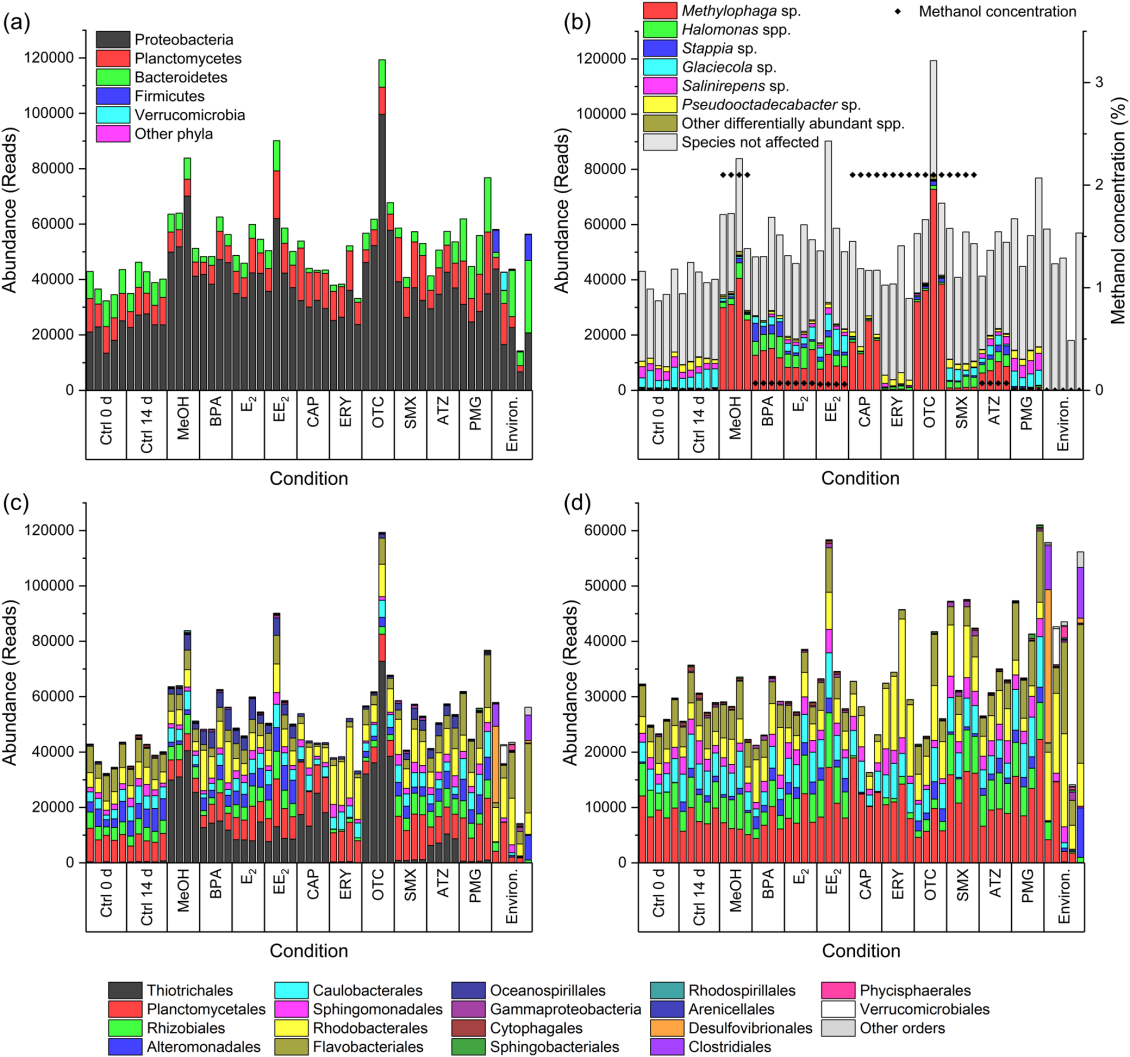
Microbiome of *Ulva compressa* upon micropollutant treatment. The cultivars of *U. compressa* were treated with micropollutants (BPA, E_2_, EE_2_, CAP, ERY, OTC, SMX, ATZ and PMG) for 14 days and compared with an environmental sample (Environ.) collected in Ria Formosa (Faro, Portugal) and controls (Ctrl 0 d, Ctrl 14 d and MeOH): (a) Absolute abundance (reads) of phyla, (b) species significantly changed in methanol controls, (c) orders and (d) orders corrected through removal of those species that were influenced by the methanol solvent. ATZ, atrazine; BPA, bisphenol A; CAP, chloramphenicol; E_2_, estradiol; EE_2_, ethinylestradiol; ERY, erythromycin; OTC, oxytetracycline; PMG, glyphosate; SMX, sulfamethoxazole.

To assess the general trends and major differences, we compared the effects of all the treatments at the phylum and order levels (Figure 3). The microbiome associated with *Ulva* predominantly comprised members of the phyla Proteobacteria, Planctomycetes, Bacteroidetes and Firmicutes. These groups, dominated by Proteobacteria, represent the core microbial constituents and were consistently detected across samples (Figure 3a). The microbiome composition of the environmental samples from all treated samples of the cultivar clearly varied (Figure 3a–d).

Since most of the compounds were dissolved in methanol, a control comparison was conducted between samples with and without methanol (MeOH) after 14 days of incubation (Ctrl 14 d) (Figure 3b). This analysis revealed that the absolute abundance of seven ASVs increased, whereas that of five significantly decreased (*p* < 0.01). Genera such as *Methylophaga*, *Halomonas* and *Stappia* responded positively to methanol. In contrast, *Glaciecola*, *Salinirepens* and *Pseudooctadecabacter* were negatively impacted. Notably, even methanol concentrations below 0.1% influenced the microbial abundance in the treated samples (Figure 3b). Consequently, the microbial composition was analysed for all the orders (Figure 3c) and corrected for the methanol effect by removing the affected species (Figure 3d).

Since the antibiotic treatments (CAP, ERY, OTC and SMX) contained the same methanol concentration as the methanol control, they could be directly compared. The results of the ERY and SMX treatments strongly deviated from those of the methanol control (Figure 3b). For example, the ERY‐treated samples presented a lower abundance of *Rhizobiales* and *Flavobacteriales*. Moreover, the abundance of *Rhodobacterales* increased relative to that in the other samples.

CAP suppressed the growth of most microbial species that were otherwise promoted by methanol. However, *Methylophaga* sp., which thrived under methanol treatment, was only partially inhibited by CAP. In contrast, the microbial profile under OTC treatment closely resembled that under the methanol control, indicating that OTC had minimal impact compared with the other antibiotics tested (Figure 3b,c). A comparison of the effects of methanol (MeOH) treatment with those of the untreated control removed the bias introduced by MeOH and highlighted the abundance and stability of the understudied order *Planctomycetales* (Figure 3d). Overall, antibiotic treatments and methanol significantly influenced the microbial composition of *Ulva*‐associated microbiomes, with ERY, CAP and SMX causing the most pronounced shifts. Other MPs (BPA, E_2_, EE_2_, ATZ and PMG) had relatively weak effects and resembled the control sample after removing MeOH affected ASVs (Figure 3d).

### 3.5 The Core Microbiome Provides the Essential Traits of AGMPFs

**FIGURE 4:**
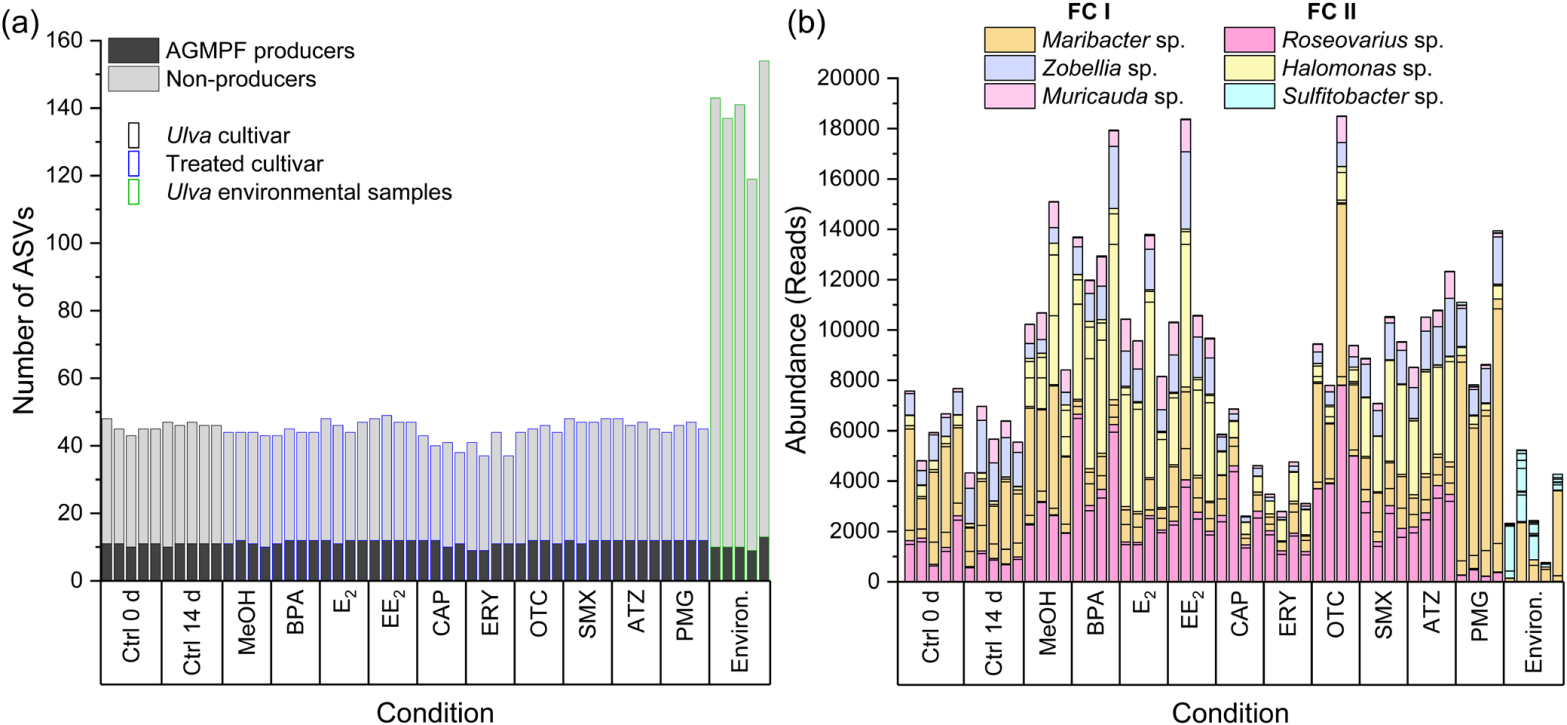
Identification of candidate bacteria that release algal growth and morphogenesis‐promoting factors (AGMPFs). (a) Laboratory control cultures (Ctrl 0 d, Ctrl 14 d, black frame) were compared with micropollutant‐treated cultures in the laboratory (blue), methanol control (MeOH), bisphenol A (BPA), estradiol (E_2_), ethinylestradiol (EE_2_), chloramphenicol (CAP), erythromycin (ERY), oxytetracycline (OTC), sulfamethoxazole (SMX), atrazine (ATZ) and glyphosate (PMG) as well as with environmental samples (green, Environ.). The numbers of ASVs are presented, divided into potential AGPMF producers (dark grey) and nonproducers (light grey), (b) absolute abundance (reads) of potential *AGPMF‐producing* species across all samples.

Finally, we categorised the microbial diversity of potential producers of AGMPFs and nonproducers within three different sample types: laboratory‐cultured samples (cultivars), antibiotic‐treated cultivars and environmental samples with their native microbiomes (Figure 4). The composition of the cultivars was relatively stable throughout the MP treatments in terms of the number of ASVs. The number of ASVs was significantly (*p* < 0.001) and distinctly smaller (41–48 ASVs) in the holobiont of the cultivar than in the environmental samples (119–154 ASVs). The total number of ASVs was smaller in the laboratory samples; the number of ASVs indicating potential AGMPF producers (9–13 ASVs) did not significantly differ among all the samples (Figure 4a). In detail, the analysis of the laboratory‐cultured samples revealed two species of *Roseovarius*, three species of *Maribacter*, three species of *Halomonas*, one species of *Muricauda* and one species of *Zobellia* (Figure 4b, Table 2, Table S3). Up to four *Maribacter* species, eight *Sulfitobacter species* and one *Muricauda* species were found in the native microbiome of the environmental samples.

**TABLE 2:** Abundance of potential ensured AGMPF‐producing species.

ASV ID	Taxonomy					Abundance in samples					
	Genus	Species	FC	Genus Conf.	Species Conf.	Cultivar (*n* = 10)		Treatments (*n* = 40)		Environment (*n* = 5)	
						Mean reads	Occurence	Mean reads	Occurence	Mean reads	Occurence
#10	*Maribacter*	*M*. *chungangensis*	I	100%	63%	2318	100%	2006	98%	768	100%
#21		*M. antarcticus*		100%	65%	609	100%	522	100%	22	20%
#41		*M*. *chungangensis*		56%	23%	103	100%	104	100%	6	20%
#49		*M. aquivivus*		95%	30%					751	100%
#16	*Zobellia*	*Z. russellii*	I	35%	10%	1197	100%	1067	100%		
#23	*Muricauda*	*M. aquimarina*	I	100%	24%	398	100%	558	100%	25	20%
#8	*Roseovarius*	*R. mucosus*	II	40%	7%	1147	100%	2599	100%		
#32		*R. tolerans*		48%	24%	103	100%	167	100%		
#11	*Halomonas*	*H. saccharevitans*	II	99%	12%	257	100%	2328	100%		
#27		*H. meridiana*		99%	14%	16	90%	390	95%		
#52		*H. axialensis*		100%	14%	4	90%	77	95%		
#56	*Sulfitobacter*	*S*. *geojensis*	II	99%	34%			8	65%	516	100%
#65		*S*. sp		88%	NA					410	100%
#84		*S. dubius*		65%	26%	8	100%	22	55%	16	80%
#88		*S. litoralis*		45%	19%					199	100%
#108		*S*. *geojensis*		55%	10%					149	100%
#125		*S. litoralis*		100%	90%					96	100%
#168		*S*. *geojensis*		60%	13%					48	100%
#170		*S. pontiacus*		38%	14%					47	100%

*Note:* The table corresponds to the Venn's diagram AGMPF circle (*n*_total_ = 19). The functional clade (FC) indicates the morphogenetic activity of identified bacteria, which have been investigated in previous studies (Grueneberg et al. 2016; Weiss et al. 2017). FC I: Induction of rhizoid and cell wall formation; FC II: Induction of growth during germination. Both functions are necessary for the entire growth and morphogenesis of *Ulva*. Confidence (Conf.) of genus and species assignment.

Bacteria releasing the *Maribacter* factor, thallusin (functional clade I, Table 2), were found in all samples, and there was substantial heterogeneity at the species level; for example, ASV #34 revealed *Maribacter chungangensis*, which can be replaced by closely related species such as *Maribacter aquivivus* or *Maribacter antarticus* (ASV #21) (Table S4). A manual NCBI‐BLAST search of the ASV sequences confirmed the identification of the genus.

Bacteria releasing the unknown *Roseovarius* factor, such as *Roseovarius*, *Sulfitobacter* or some *Halomonas* species (functional clade II, Table 2), were found in varying proportions across all the samples. The environmental samples revealed the presence of several ASVs, indicating the presence of up to eight *Sulfitobacter* strains, with *Sulfitobacter geojensis* being the most notable. The genome‐sequenced bacteria of *Halomonas* sp. MS1 and *Roseovarius* sp. MS2 could be identified via the characteristic V4 sequences ‘ASV #11’ and ‘ASV #8’, respectively (Table S4).

This study categorised microbial diversity according to potential AGMPF producers and nonproducers across laboratory‐cultured, antibiotic‐treated and environmental samples. While the cultivars presented significantly fewer ASVs than the environmental samples did, the number of AGMPF‐producing ASVs remained consistent, highlighting their role as key indicators of the *Ulva* core microbiome.

### 3.6 Temporal Shifts in the *Ulva compressa* Microbiome During MP Exposure

**FIGURE 5:**
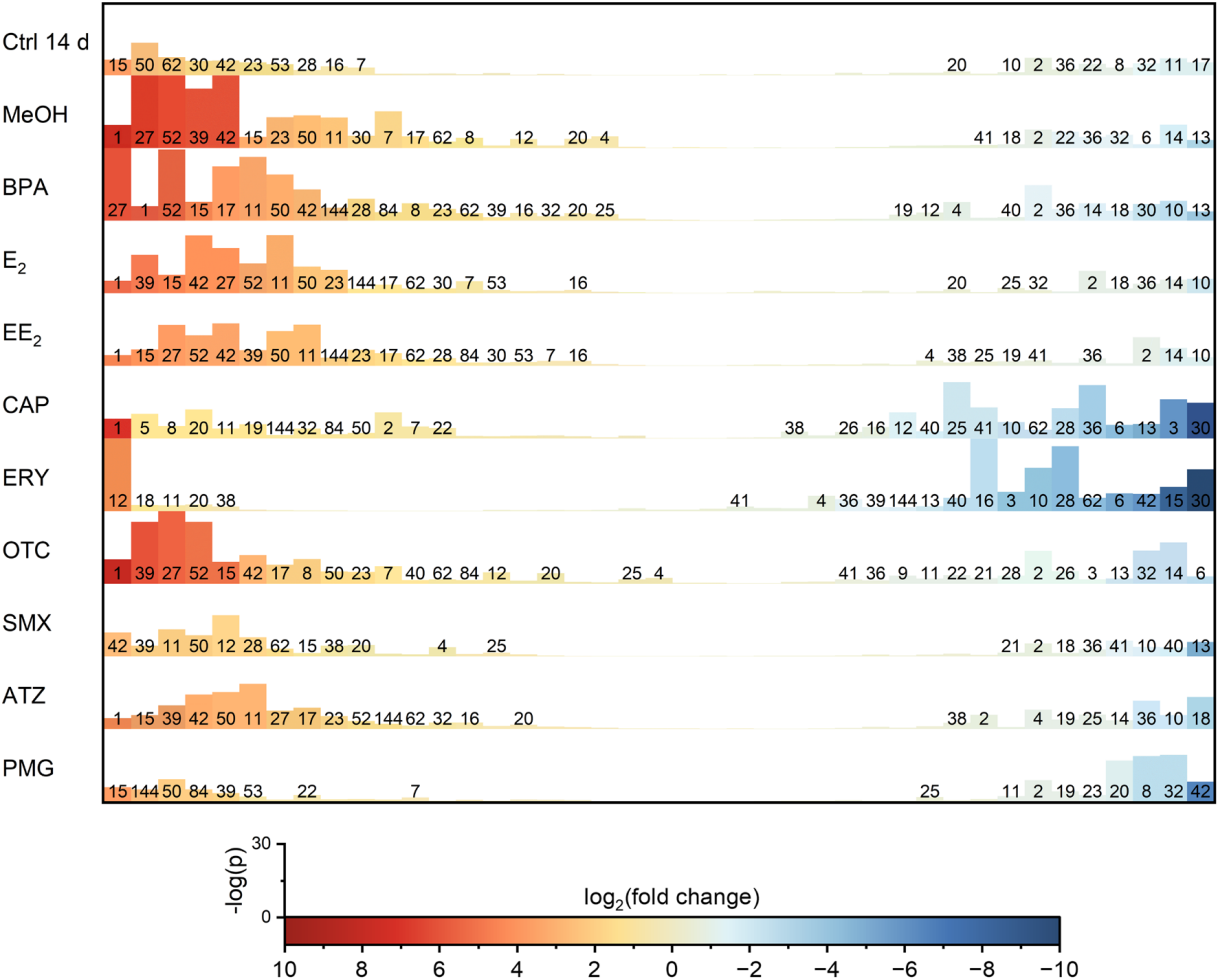
Abundancy of the ASVs in the microbiome of *Ulva compressa* after treatment. Each bar represents one ASV and includes the associated number when a significant change over time was present. The bar height represents statistical confidence, and the colour indicates the log_2_‐fold change compared with the control before 14 days of incubation. Red indicates an increase in ASV abundance, whereas blue represents a decrease (*n* = 4–5). ATZ, atrazine; BPA, bisphenol A; CAP, chloramphenicol; Ctrl d 0, control before incubation; Ctrl d 14, control after 14 days of incubation; E_2_, estradiol; EE_2_, ethinylestradiol; ERY, erythromycin; MeOH, methanol control; OTC, oxytetracycline; PMG, glyphosate; SMX, sulfamethoxazole.

All the treatments with MPs were evaluated against the control before 14 days of incubation to identify the differentially abundant strains (Figure 5). The heatmap illustrates the differential presence of the 42 ASVs in the cultivar microbiome. The CAP, OTC and ERY treatments resulted in the most pronounced changes, with strong upregulation in some ASVs and significant downregulation in others. ATZ, BPA and estrogenic compounds (E_2_, EE_2_) exhibited moderate shifts, whereas MeOH and PMG had weaker effects. Certain ASVs (e.g., #1, 15 and 35) were frequently upregulated across treatments, whereas others (e.g., #2, 4 and 10) were consistently downregulated, suggesting treatment‐specific regulatory effects (Figure 5). Overall, after 14 days of incubation, slight microbiome alterations were observed, while AGMPF‐producing bacteria (ASV #8, 11, 16, 21, 23, 27 and 32), including *Maribacter* sp., *Halomonas* sp. or *Roseovarius* sp. remained resilient to antibiotic treatments.

At the species level, bacteria that release AGMPFs and phenocopy *Roseovarius* sp. MS2 (e.g., ASVs #8 and #11, Table S3) were often more prominent than others, indicating their functional significance and resilience to antibiotic treatments. Potential thallusin producers phenocopying *Maribacter* sp. MS6 neither gained nor lost abundance in the microbiome.

**FIGURE 6:**
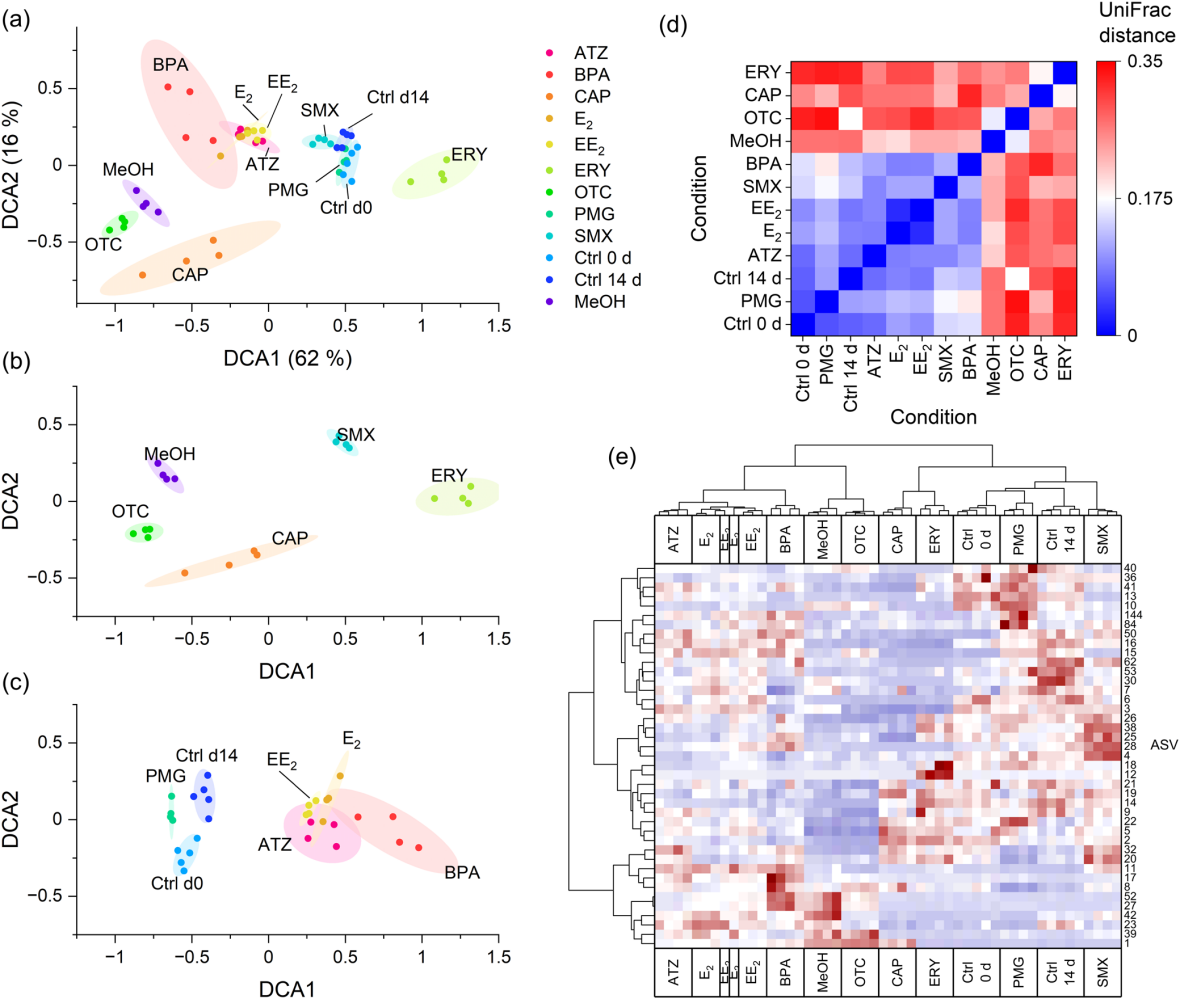
Multivariate analysis after the addition of micropollutants. (a) Detrended correspondence analysis of controls and treated cultures for all samples, (b) samples with 2.1% methanol and (c) samples with less than 0.1% methanol with 90% confidence ellipses. (d) Quantification of the dissimilarity between different treatments (UniFrag distance). (e) The cluster heatmap visualises higher (red) or lower (blue) abundances of 42 ASVs depending on the treatment (*n =* 4–5). ATZ, atrazine; BPA, bisphenol A; CAP, chloramphenicol; Ctrl d 0, control before incubation; Ctrl d 14, control after 14 days of incubation; E_2_, estradiol; EE_2_, ethinylestradiol; ERY, erythromycin; MeOH, methanol control; OTC, oxytetracycline; PMG, glyphosate; SMX, sulfamethoxazole.

Detrended correspondence and UniFrag analyses revealed distinct clustering of CAP, ERY and OTC, with OTC closely aligning with the methanol control, while SMX and PMG clustered near the controls. The correlation analysis highlighted differences in treatment relationships, particularly between SMX and PMG (Figure 6). In a DCA of all the methanol control treatments, CAP, ERY and OTC formed individual clusters (Figure 6a). The OTCs clustered closely to those in the methanol control group. Moreover, two antibiotics, CAP and ERY, presented the most pronounced differences. The four clusters of the two controls, SMX and PMG, were very closely related. PMG clustered closer to the control before incubation than after.

Considering the samples with identical methanol concentrations underscores their distinctions. For example, this approach differentiates OTC from the methanol control group (MeOH) (Figure 6b). The treatments containing negligible quantities of methanol (< 0.07%), however, exhibited considerable divergence from the controls on days 0 and 14, although the variations were minor (EE_2_, E_2_, ATZ and BPA) (Figure 6c).

The UniFrac distance matrix shed light on the differences between microbial communities subjected to different chemical treatments and control conditions. The lowest distance values in the matrix occurred between E_2_ and EE_2_ (0.02961), indicating that these two treatments resulted in highly similar microbial community structures. Their distances to the control samples were also relatively low, suggesting that both E_2_ and EE_2_ exert only minor changes on the microbiome. Another low‐distance pair was PMG and Ctrl 0 d (0.05629), indicating that, compared with the untreated baseline, PMG had a minimal or possibly retaining effect on the microbial composition (Figure 6d).

In contrast, some treatments presented high UniFrac distances from control samples and other treatments, suggesting a more profound impact on the microbial community structure. For example, ERY showed high dissimilarities, such as 0.32 from Ctrl 0 d, indicating a significant alteration in microbiota. CAP and MeOH also presented relatively high distances from the controls and clustered with OTC and ERY, suggesting comparable disruptive effects. Compounds such as ATZ, SMX and BPA exhibited intermediate levels of divergence. These treatments were moderately distant from both the control samples and each other, placing them between the low‐impact and high‐impact clusters. Taken together, the UniFrac distance matrix findings suggest three broad groupings based on microbiome similarity. The first group included PMG, E_2_, EE_2_ and the control samples, which were characterised by minimal microbiome disruption. The second group, ATZ, SMX and BPA, presented moderate changes. The third group, comprising OTC, ERY, CAP and MeOH, presented the greatest divergence, likely exerting the most substantial impacts on the microbial community composition (Figure 6d).

This clustering implies varying degrees of microbial perturbation across treatments, possibly reflecting different modes of action or toxicity levels. These observations provide a foundation for further exploration using hierarchical clustering to better visualise the underlying patterns in microbial community responses.

All the treatments were grouped distinctly in the clustered heatmap except for E_2_ and EE_2_ (Figure 6e), which exhibited insufficient variation for a clear distinction.

Hierarchical clustering revealed four distinct groups among the treatments. The first cluster comprised ATZ, E_2_ and EE_2_, which grouped closely together. Their microbial profiles remained near baseline conditions, aligning well with the low UniFrac distances observed between these treatments and the controls. Another central cluster was CAP and ERY, which are associated with more extensive changes in microbial composition. The third cluster comprised the two control samples (Ctrl 0 d, Ctrl 14 d) and PMG, which are tightly grouped, reflecting stable and less perturbed microbial communities. Notably, SMX appeared adjacent to the control group but remained slightly separate, suggesting a moderate impact on the microbial structure.

Several ASVs clustered and exhibited strong enrichment, indicating a shared response pattern to specific treatments. For example, ASVs 10, 13, 36, 40, 41, 84 and 144 were notably enriched (deep red), including e.g., *Maribacter* (ASV 10), in the PMG treatment but were consistently depleted across all other conditions. These findings suggest that these ASVs may be selectively stimulated by components unique to the PMG treatment. Similarly, a distinct cluster of ASVs 4, 25, 26, 28 and 38 (see for the taxa Table S4) showed pronounced enrichment in the SMX treatment group, highlighting a treatment‐specific microbial signature. The clear clustering of these ASVs highlights the consistent and differential impacts of individual compounds on the microbial community structure.

## 4 Discussion

Our study provides insights into the effects of long‐term cultivation and MP exposure on the microbiome of *Ulva*, exemplified by the model species *U. compressa* . The results revealed significant shifts in microbial diversity under laboratory conditions and upon exposure to various environmental contaminants, emphasising the resilience of key microbial taxa and their functional roles in algal holobionts.

The shared core microbiome comprises 20 species. A substantial proportion (20%–25%) of these bacteria play a pivotal role in facilitating algal development through AGMPFs and are, thus, part of the functional core microbiome. Spoerner et al. (2012) isolated AGMPF‐releasing bacteria from these core bacteria. Importantly, as AGMPFs such as thallusin are highly biologically active, even a reduced population of producer strains is sufficient to promote algal growth and morphogenesis (Ulrich et al. 2022).

### 4.1 Impact of Long‐Term Cultivation on Microbiome Diversity

There is an ongoing and engaging debate about whether *Ulva*‐associated bacteria are assembled stochastically (Burke et al. 2011), selectively attracted by specific chemoattractants and nutrients (Kessler et al. 2018), or shaped by a combination of both mechanisms. Despite the pioneering exploitative microbiome analyses by Burke et al. (2011), studies of coastal‐located aquacultures (Califano et al. 2020; de Jager et al. 2024; van der Loos et al. 2024), and extensive field experiments (van der Loos et al. 2023; Mudlaff et al. 2025), there are few hypothesis‐driven approaches that address this question in *Ulva* (Ghaderiardakani et al. 2017; Kessler et al. 2018).

Here, we defined key core microbiome members as ASVs present in both long‐term cultivar and environmental samples, identifying 20 shared ASVs that met these criteria (Figure 1, Table 1).

The cultivar harboured only 48 ASVs, in stark contrast with the approximately 200 ASVs observed in the native samples, which are within the previously published range (e.g., van der Loos et al. 2023). These findings suggest that long‐term laboratory cultivation selects for a stable but less diverse core microbiome (Figure 1). While the cultivar microbiome remains functionally viable, it differs from the more diverse and dynamic microbiomes observed in natural environments. Beta diversity analysis further supported these findings, demonstrating a clear separation between laboratory and environmental samples. The clustering pattern of laboratory and MP‐treated samples highlights the intrinsic stability of the cultivated microbiome, with only minor alterations upon exposure to contaminants (Figures 1 and 6).

### 4.2 Resilience of the Core Microbiome and AGMPF Production

AGMPF production has been demonstrated in previous studies involving the inoculation of axenic cultures of *U. compressa* with core isolates of identified strains with essential functional roles (Spoerner et al. 2012; Grueneberg et al. 2016; listed in Table 2). Among the 20 shared ASVs were the known symbiotic strains that support *Ulva* by releasing AGMPFs in exchange for nutrients. These ASVs have been reported in various microbiome studies of *Ulva* and environmental conditions (Califano et al. 2020; Nguyen et al. 2023; van der Loos et al. 2023, 2024; Ghaderiardakani et al. 2024); however, the functional roles of most of the bacteria remain unknown and should be further investigated.

Since the number of potential AGMPF‐producing ASVs and, therefore, the number of commensal symbionts of *Ulva* did not significantly differ between samples, long‐term cultivation clearly results in the loss of most (redundant) strains while maintaining those that live in symbiosis with *Ulva*. In contrast, despite overall microbiome alterations, the core microbial taxa associated with *Ulva*, particularly those involved in the release of AGMPFs, remained largely resilient (Figure 4). Only a few taxa can produce and release thallusin, indicating the potential unique biosynthesis mechanisms involved in this process (Le et al. 2019). These genera are mandatory for the growth and morphogenesis of *Ulva*. While cultivars presented a significantly lower total number of ASVs than environmental samples, the number of AGMPF‐producing ASVs remained consistent (Table 2, Figure 4a), and the abundance increased (Figure 4b). These findings highlight the importance of these microbial traits in sustaining *Ulva* growth and development.

Notably, the functional role of specific bacteria within the holobiont can shift from beneficial to detrimental under stress conditions, a phenomenon known as the ‘Jekyll and Hyde’ effect (Seyedsayamdost et al. 2011; Hmani et al. 2024). While metagenomic analyses represent a valuable step forward, they may still fail to capture these dynamic functional changes. Moreover, traits of the native microbiome could balance the adverse or less beneficial effects of specific bacteria in a stress situation through the alternative traits of multiple bacterial resources (Hmani et al. 2024). Regardless, microbiome analysis must be accompanied by functional biotests and molecular biological investigations. Interestingly, additional probiotic effects on *Ulva* growth in multitrophic aquaculture have been recently described upon the inoculation of *Phaeobacter* sp., which releases the multifunctional antibiotic tropodithietic acid (Lindqvist et al. 2023; Qui‐Minet et al. 2025; Del Olmo et al. 2025).

### 4.3 Symbiotic Bacteria Do Not Contribute to the Bioremediation of MPs

*Ulva compressa* has been shown to remove several MPs, particularly phenolic compounds such as bisphenols and estradiols, through degradation upon uptake (Hardegen et al. 2025). Although the two symbiotic strains *Roseovarius* sp. MS2 and *Maribacter* sp. MS6 were unable to remove these compounds, it was hypothesised that other symbiotic strains might be capable of doing so, thereby enhancing *Ulva*'s already high bioremediation potential even further. However, the absence of differences in MP removal between the previously published tripartite *Ulva* community (*Ulva*‐*Roseovarius*‐*Maribacter*) and the cultivar containing more than 40 bacterial strains (Hardegen et al. 2023) (Figure 2b) suggests that *Ulva*'s core microbiome does not contribute to this process. These findings highlight the selection of strains that benefit *Ulva* during long‐term cultivation. However, these strains cannot metabolise newly introduced compounds due to a lack of selective pressure. Conversely, we hypothesise that *Ulva* specimens exposed to a constant influx of MPs are likely to harbour bacterial symbionts that can remove these compounds. To identify these symbiotic bacterial species, sampling should be conducted at contaminated sites, which is a strategy commonly employed for the isolation of MP‐degrading free‐living bacteria.

### 4.4 Effects of MPs on Microbiome Composition

The exposure of *Ulva*‐associated microbiomes to common antibiotics, hormonal disruptors and pesticides revealed distinct alterations in microbial composition. Although *Ulva* can rapidly absorb various MPs, such as hormonal disruptors and pesticides, its growth and development are often unaffected (Trinelli et al. 2013; Areco et al. 2021; Hardegen et al. 2023), whereas its associated microbiome undergoes slight alterations (Figure 6d).

In contrast, antibiotic exposure led to more significant microbiome changes, with several species declining in abundance (Figure 5), although none disappeared entirely after 14 days of exposure. This finding further illustrates that antibiotics do not eliminate bacteria and are ineffective in preparing axenic cultures (Wichard 2015). However, antibiotics can profoundly shape the microbiome's ecology, causing lasting changes to the host (e.g., Langdon et al. 2016). After 14 days of MP exposure, minimal alterations were observed in the core microbiome, with AGMPF‐producing bacteria exhibiting resistance to antibiotic treatments. CAP and ERY caused significant reductions in certain ASVs, whereas SMX induced the smallest changes (Figure 5). The results of the correspondence analysis and UniFrag clustering further revealed distinct treatment effects, with CAP, ERY and OTC forming separate clusters, whereas SMX and PMG closely aligned with the controls. Notably, methanol strongly influenced microbial clustering, reinforcing the need to account for solvent effects when interpreting MP‐induced changes. DMSO and methanol are solvents commonly used in in vitro experiments, particularly when low‐soluble compounds are introduced into a cellular system (Inoue 2011; Dyrda et al. 2019). In our study, the added methanol can be used as an energy source through oxidation to CO_2_ and as a carbon source that is assimilated into biomass by methylotrophs (Sun et al. 2011). Indeed, methanol significantly influences microbial abundance, with specific genera such as *Methylophaga* thriving under methanol treatment (Janvier and Gasser 1987), whereas *Glaciecola*, *Salinirepens*, and *Pseudooctadecabacter* are negatively impacted. These findings indicate that even trace amounts of methanol (< 0.07%) can influence microbial community structures and must be accounted for when analysing pollutant effects; however, methanol does not hamper the core functions of the microbiome under the selected laboratory conditions.

Marine bacteria have naturally developed antibiotic resistance through evolutionary adaptation, environmental pressures, genetic exchange and biofilm formation (Krupesha Sharma and Sumithra 2023). Among the antibiotics tested, only ERY and CAP caused pronounced shifts in microbiome composition, significantly reducing the abundance of *Rhizobiales* and *Flavobacteriales*. In contrast, OTC had a relatively mild impact, presumably because it is unstable under culture conditions (Hardegen et al. 2023). Compared with other antibiotics, ERY and CAP are potent against marine bacteria because they target protein synthesis, have broad‐spectrum activity, and exhibit relatively lower levels of preexisting resistance in marine bacteria (Hatosy and Martiny 2015). Importantly, the impact of human activities spreads antibiotic resistance genes in marine microbial populations, for example, those near sewage discharge areas (Griffin et al. 2020). Therefore, increasing antibiotic pollution in marine environments might lead to increased resistance, making monitoring antibiotic effectiveness crucial. Our findings imply that even highly contaminated wastewater close to sewage outfall systems might not harm the microbiome‐promoted development of *Ulva*; however, more extensive studies are necessary.

Other pollutants, such as BPA, E_2_ and EE_2_, exerted comparatively weak effects, probably due to their rapid removal through degradation or sorption by *Ulva* (Hardegen et al. 2023; Ludmila et al. 2023). These findings suggest that antibiotic treatments exert intense selective pressures on the *Ulva* microbiome, whereas other MPs, including hormonal disruptors, induce more subtle compositional shifts. Future studies will explore which MPs cause long‐term effects on *Ulva* growth, the associated microbiome and how toxic compounds might synergistically trigger these effects. In this study, none of the treatments impaired the production of thallusin, as evidenced by the absence of cell wall protrusions, a phenotype typically associated with thallusin deficiency (Spoerner et al. 2012).

### 4.5 Limitations of the Study

While this study of the *Ulva* cultivar microbiome provides important insights into host–microbe interactions, some limitations must be recognised. While long‐term laboratory cultures are regulated and reproducible, they cannot adequately represent the diversity and dynamics of natural microbial communities. Selective pressures in laboratory conditions and treatment can result in the extinction of environmentally sensitive species, thereby altering key microbiome traits. Furthermore, environmental samples introduce heterogeneity due to shifting abiotic factors, seasonal variations and stochastic microbial colonisation processes, complicating cross‐habitat and robust comparisons. Furthermore, distinguishing between transient and functionally integrated bacteria (into the holobiont) remains challenging, especially when employing amplicon‐based techniques with limited strain resolution. While comparing laboratory and field samples provides a helpful framework for identifying stable and responsive microbial traits, it is essential to exercise caution when generalising results across different ecological situations. To overcome these restrictions, multiomics data must be integrated, particularly transcriptomics, to decipher environment‐dependent gene expression in bacteria. Furthermore, sampling must be expanded over time and space. However, at present, predictive metagenomics or functional annotations cannot adequately capture the mechanisms of morphogenetic promotion, as these processes appear to involve novel, hormone‐like compounds and biosynthetic pathways that remain unknown. In our model system of *Ulva* and its associated bacteria, these compounds play a crucial role in morphogenesis, yet their synthesis and activation pathways have not been identified.

Furthermore, a significant impact of methanol on the microbiome was observed. While the effect of methanol was factored in using controls and statistical analysis, any synergistic or anti‐synergistic effects of methanol and MPs could not be accounted for. Consequently, if the experiment was repeated without adding methanol alongside the MPs, the findings might differ.

## 5 Conclusion

Our study demonstrates that long‐term cultivation and exposure to MPs have profound effects on the *Ulva* holobiont. In particular, we identified four key outcomes:

- long‐term cultivation drastically reduces microbiome diversity,
- the resulting ‘core’ microbiome remains resilient to chemical stressors and continues to support algal growth,
- antibiotics are ineffective for axenization of *Ulva*, and
- *Ulva* is capable of removing endocrine disruptors, potentially reducing stress in fish aquaculture.

Despite cultivation and MP treatment changes, key AGMPF‐producing taxa remain resilient, underscoring their ecological significance within *Ulva* holobionts. This further demonstrates that antibiotics are ineffective for removing bacteria when aiming to conduct morphogenetic studies. Nonetheless, *Ulva* efficiently eliminates steroid hormones from growth media under continuous microbiota conditions, highlighting its potential use in polluted waters or integrated multitrophic aquaculture systems where fish‐derived hormones may accumulate and affect ecosystem dynamics. Future research should explore the functional implications of these microbial changes, notably for algal growth, stress resistance and environmental adaptability. Understanding the complicated interactions between *Ulva* and its microbiome will provide insight into the wider ecological implications of anthropogenic contamination for marine microbial ecosystems.

## Author Contributions

**Justus Hardegen:** conceptualization, formal analysis, investigation, validation, visualization, writing – original draft. **Gabriel Amend:** investigation. **Thomas Wichard:** conceptualization, formal analysis, funding acquisition, resources, supervision, validation, writing – review and editing. All authors have read and agreed to the published version of the manuscript.

## Ethics Statement

All prevailing regulations and conventions, and normal scientific ethical practices, have been respected.

## Conflicts of Interest

The authors declare no conflicts of interest.

## Supporting information


**Table S1:** Experiment parameters for the addition of chemical stressors.
**Table S2:** Genbank accession numbers. Samples as part of the BioProject PRJNA828511 (NCBI).
**Table S3:** Overview of the determined amplicon sequence variances in various treatments. Number of replicates reads (average of replicates), maximum observed ASVs and alpha diversity (average of replicates) for lab cultures, treated samples and five environmental samples.
**Figure S1:** Rarefraction curves of bacterial richness, Shannon and Simpson. Replicates within each treatment displayed consistent profiles, indicating good reproducibility of the sequencing data. Control samples exhibited higher or similar diversity as pollutant‐exposed samples. Antibiotic treatments, particularly erythromycin (ERY), chloramphenicol (CAP) and oxytetracycline (OTC), caused the most substantial reductions. Treatments with endocrine disruptors (BPA, E_2_ and EE_2_) showed intermediate effects on microbial richness and diversity. In contrast, environmental samples (Ria Formosa: RFM #4–8) displayed substantially higher richness compared to long‐term cultured *Ulva*, highlighting the overall reduction of microbiome diversity under laboratory cultivation.

**Table S4:** Summary of the phenotypes of the identified bacterial species and the ASV sequences used for the microbiome analysis.

## Data Availability

The data that support the findings of this study are openly available in NCBI Sequence Read Archive at https://pubmed.ncbi.nlm.nih.gov/, reference number PRJNA828511, which includes the BioSample accessions numbers SAMN48728450–SAMN48728504.

## References

[emi470230-bib-0001] Allard, P. 2014. “Chapter 27–Bisphenol A.” In Biomarkers in Toxicology, edited by R. C. Gupta , 459–474. Academic Press.

[emi470230-bib-0087] Alsufyani, T. , A. H. Engelen , O. E. Diekmann , S. Kuegler , and T. Wichard . 2014. “Prevalence and Mechanism of Polyunsaturated Aldehydes Production in the Green Tide Forming Macroalgal Genus Ulva (Ulvales, Chlorophyta).” Chemistry and Physics of Lipids 183: 100–109. 10.1016/j.chemphyslip.2014.05.008. 24915501

[emi470230-bib-0002] Apprill, A. , S. McNally , R. Parsons , and L. Weber . 2015. “Minor Revision to V4 Region Ssu rRNA 806r Gene Primer Greatly Increases Detection of Sar11 Bacterioplankton.” Aquatic Microbial Ecology 75: 129–137.

[emi470230-bib-0003] Areco, M. M. , V. N. Salomone , and M. S. Afonso . 2021. “ *Ulva lactuca *: A Bioindicator for Anthropogenic Contamination and Its Environmental Remediation Capacity.” Marine Environmental Research 171: 105468. 34507027 10.1016/j.marenvres.2021.105468

[emi470230-bib-0004] Barnett, J. A. , and D. L. Gibson . 2020. “Separating the Empirical Wheat From the Pseudoscientific Chaff: A Critical Review of the Literature Surrounding Glyphosate, Dysbiosis and Wheat‐Sensitivity.” Frontiers in Microbiology 11: 556729. 33101230 10.3389/fmicb.2020.556729 PMC7545723

[emi470230-bib-0005] Blomme, J. , T. Wichard , T. B. Jacobs , and O. De Clerck . 2023. “ *Ulva*: An Emerging Green Seaweed Model for Systems Biology.” Journal of Phycology 59: 433–440. 37256696 10.1111/jpy.13341

[emi470230-bib-0006] Burke, C. , P. Steinberg , D. Rusch , S. Kjelleberg , and T. Thomas . 2011. “Bacterial Community Assembly Based on Functional Genes Rather Than Species.” Proceedings of the National Academy of Sciences of the United States of America 108: 14288–14293. 21825123 10.1073/pnas.1101591108 PMC3161577

[emi470230-bib-0007] Califano, G. , M. Kwantes , M. H. Abreu , R. Costa , and T. Wichard . 2020. “Cultivating the Macroalgal Holobiont: Effects of Integrated Multi‐Trophic Aquaculture on the Microbiome of *Ulva* *rigida* (Chlorophyta).” Frontiers in Marine Science 7: 52.

[emi470230-bib-0008] Califano, G. , and T. Wichard . 2018. “Chapter 9. Preparation of Axenic Cultures in *Ulva* (Chlorophyta).” In Protocols for Macroalgae Research, edited by B. Charrier , T. Wichard , and C. R. K. Reddy . CRC Press.

[emi470230-bib-0009] Chen, J. M. , R. X. Sun , C. G. Pan , Y. Sun , B. X. Mai , and Q. X. Li . 2020. “Antibiotics and Food Safety in Aquaculture.” Journal of Agricultural and Food Chemistry 68: 11908–11919. 32970417 10.1021/acs.jafc.0c03996

[emi470230-bib-0010] Cole, J. R. , Q. Wang , J. A. Fish , et al. 2014. “Ribosomal Database Project: Data and Tools for High Throughput rRNA Analysis.” Nucleic Acids Research 42: D633–D642. 24288368 10.1093/nar/gkt1244 PMC3965039

[emi470230-bib-0011] Das, S. , N. M. Ray , J. Wan , A. Khan , T. Chakraborty , and M. B. Ray . 2017. “Micropollutants in Wastewater: Fate and Removal Processes.” In Physico‐Chemical Wastewater Treatment and Resource Recovery, edited by R. Farooq and Z. Ahmad , 74–107. Intech Open.

[emi470230-bib-0012] De Clerck, O. , S. M. Kao , K. A. Bogaert , et al. 2018. “Insights Into the Evolution of Multicellularity From the Sea Lettuce Genome.” Current Biology 28: 2921–2933. 30220504 10.1016/j.cub.2018.08.015

[emi470230-bib-0013] de Jager, K. , M. Brink‐Hull , J. J. Bolton , M. D. Cyrus , and B. M. Macey . 2024. “Bacterial Microbiome Dynamics in Commercial Integrated Aquaculture Systems Growing *Ulva i*n Abalone Effluent Water.” Journal of Applied Phycology 36: 2823–2849.

[emi470230-bib-0086] Del Olmo, G. , P. Ruiz , J. Nappi , et al. 2025. “Optimizing Ulva‐Phaeobacter co‐culture: A Two‐Phase Light Intensity Approach for Integrated Multi‐Trophic Aquaculture Applications.” Journal of Applied Phycology 37: 1227–1240. 10.1007/s10811-025-03461-9.

[emi470230-bib-0014] Dyrda, G. , E. Boniewska‐Bernacka , D. Man , K. Barchiewicz , and R. Słota . 2019. “The Effect of Organic Solvents on Selected Microorganisms and Model Liposome Membrane.” Molecular Biology Reports 46: 3225–3232. 30937654 10.1007/s11033-019-04782-y

[emi470230-bib-0015] Edgar, R. C. 2010. “Search and Clustering Orders of Magnitude Faster Than Blast.” Bioinformatics 26: 2460–2461. 20709691 10.1093/bioinformatics/btq461

[emi470230-bib-0016] Edgar, R. C. 2016a. “Unoise2: Improved Error‐Correction for Illumina 16S and Its Amplicon Sequencing.” *bioRxiv* . 10.1101/081257.

[emi470230-bib-0017] Edgar, R. C. 2016b. “Sintax: A Simple Non‐Bayesian Taxonomy Classifier for 16S and Its Sequences.” *bioRxiv* . 10.1101/074161.

[emi470230-bib-0018] Edgar, R. C. 2018. “Uncross2: Identification of Cross‐Talk in 16s rRNA OTU Tables.” *bioRxiv* . 10.1101/400762.

[emi470230-bib-0019] El Semary, N. 2023. “Use of Algae in Wastewater Treatment.” In Recent Trends in Constructed Wetlands for Industrial Wastewater Treatment, edited by M. P. Shah , 161–176. Springer Nature.

[emi470230-bib-0020] Føyn, B. 1958. “Über die Sexualität und den Generationswechsel von *Ulva mutabilis* .” Archiv für Protistenkunde 102: 473–480.

[emi470230-bib-0021] Gavrilescu, M. , K. Demnerova , J. Aamand , S. Agathoss , and F. Fava . 2015. “Emerging Pollutants in the Environment: Present and Future Challenges in Biomonitoring, Ecological Risks and Bioremediation.” New Biotechnology 32: 147–156. 24462777 10.1016/j.nbt.2014.01.001

[emi470230-bib-0022] Ghaderiardakani, F. , G. Califano , J. F. Mohr , M. H. Abreu , J. C. Coates , and T. Wichard . 2019. “Analysis of Algal Growth‐ and Morphogenesis‐Promoting Factors in an Integrated Multi‐Trophic Aquaculture System for Farming *Ulva* spp .” Aquaculture Environment Interactions 11: 375–391.

[emi470230-bib-0023] Ghaderiardakani, F. , J. C. Coates , and T. Wichard . 2017. “Bacteria‐Induced Morphogenesis of *Ulva* *intestinalis* and *Ulva* *mutabilis* (Chlorophyta): A Contribution to the Lottery Theory.” FEMS Microbiology Ecology 93: fix094. 28810708 10.1093/femsec/fix094 PMC5812546

[emi470230-bib-0024] Ghaderiardakani, F. , L. Langhans , V. B. Kurbel , S. Fenizia , and T. Wichard . 2022. “Metabolite Profiling Reveals Insights Into the Species‐Dependent Cold Stress Response of the Green Seaweed Holobiont *Ulva* (Chlorophyta).” Environmental and Experimental Botany 200: 104913.

[emi470230-bib-0025] Ghaderiardakani, F. , J. F. Ulrich , E. Barth , M. L. Quartino , and T. Wichard . 2024. “Algal Growth and Morphogenesis‐Promoting Factors Released by Cold‐Adapted Bacteria Contribute to the Resilience and Morphogenesis of the Seaweed *Ulva* (Chlorophyta) in Antarctica (Potter Cove).” Journal of Plant Growth Regulation. 10.1007/s00344-024-11507-4.

[emi470230-bib-0026] Griffin, D. W. , K. Banks , K. Gregg , S. Shedler , and B. K. Walker . 2020. “Antibiotic Resistance in Marine Microbial Communities Proximal to a Florida Sewage Outfall System.” Antibiotics 11: 118. 10.3390/antibiotics9030118 PMC7148511 32168949

[emi470230-bib-0027] Grueneberg, J. , A. H. Engelen , R. Costa , and T. Wichard . 2016. “Macroalgal Morphogenesis Induced by Waterborne Compounds and Bacteria in Coastal Seawater.” PLoS One 11: e0146307. 26745366 10.1371/journal.pone.0146307 PMC4720170

[emi470230-bib-0028] Hardegen, J. , G. Amend , and T. Wichard . 2023. “Lifecycle‐Dependent Toxicity and Removal of Micropollutants in Algal Cultures of the Green Seaweed *Ulva* (Chlorophyta).” Journal of Applied Phycology 35: 2031–2048.

[emi470230-bib-0029] Hardegen, J. , P. Braeutigam , C. Abendroth , and T. Wichard . 2021. “Bisphenol A: Quantification in Complex Matrices and Removal by Anaerobic Sludges.” Pollutants 1: 194–206.

[emi470230-bib-0030] Hardegen, J. B. , M. S. F. Knips , J. K. Däumer , S. Kretzer , and T. Wichard . 2025. “Biodegradation of Xenoestrogens by the Green Tide Forming Seaweed *Ulva*: A Model System for Bioremediation.” ACS ES&T Water 5: 1195–1206. 40110440 10.1021/acsestwater.4c00961 PMC11915382

[emi470230-bib-0031] Hatosy, S. M. , and A. C. Martiny . 2015. “The Ocean as a Global Reservoir of Antibiotic Resistance Genes.” Applied and Environmental Microbiology 81: 7593–7599. 26296734 10.1128/AEM.00736-15 PMC4592852

[emi470230-bib-0032] Hmani, I. , F. Ghaderiardakani , L. Ktari , M. E. Bour , and T. Wichard . 2024. “High‐Temperature Stress Induces Bacteria‐Specific Adverse and Reversible Effects on *Ulva* (Chlorophyta) Growth and Its Chemosphere in a Reductionist Model System.” Botanica Marina 67: 131–138.

[emi470230-bib-0033] Höck, M. , and S. Ziesing . 2020. “Tetracycline (Doxycyclin) und Glycylcycline.” In Medizinische Mikrobiologie und Infektiologie, edited by S. Suerbaum , G.‐D. Burchard , S. H. E. Kaufmann , and T. F. Schulz , 979–981. Springer.

[emi470230-bib-0034] Inoue, A. 2011. “Diversity and Ecology of Organic Solvent Tolerant Microorganisms.” In Extremophiles Handbook, edited by K. Horikoshi , 945–970. Springer Japan.

[emi470230-bib-0035] Janvier, M. , and F. Gasser . 1987. “Purification and Properties of Methanol Dehydrogenase From *Methylophaga marina* .” Biochimie 69: 1169–1174. 3129021 10.1016/0300-9084(87)90143-x

[emi470230-bib-0036] Kessler, R. W. , A. Weiss , S. Kuegler , C. Hermes , and T. Wichard . 2018. “Macroalgal‐Bacterial Interactions: Role of Dimethylsulfoniopropionate in Microbial Gardening by *Ulva* (Chlorophyta).” Molecular Ecology 27: 1808–1819. 29290092 10.1111/mec.14472

[emi470230-bib-0037] Krupesha Sharma, S. R. , and T. G. Sumithra . 2023. “Antimicrobial Resistance in Marine Ecosystem: An Emerging Threat for Public Health.” In Handbook on Antimicrobial Resistance: Current Status, Trends in Detection and Mitigation Measures, edited by M. P. Mothadaka , M. Vaiyapuri , M. Rao Badireddy , C. Nagarajrao Ravishankar , R. Bhatia , and J. Jena , 67–94. Springer Nature Singapore.

[emi470230-bib-0038] Kummerer, K. 2009. “Antibiotics in the Aquatic Environment–A Review–Part I.” Chemosphere 75: 417–434. 19185900 10.1016/j.chemosphere.2008.11.086

[emi470230-bib-0039] Langdon, A. , N. Crook , and G. Dantas . 2016. “The Effects of Antibiotics on the Microbiome Throughout Development and Alternative Approaches for Therapeutic Modulation.” Genome Medicine 8: 39. 27074706 10.1186/s13073-016-0294-z PMC4831151

[emi470230-bib-0040] Laurenson, J. P. , R. A. Bloom , S. Page , and N. Sadrieh . 2014. “Ethinyl Estradiol and Other Human Pharmaceutical Estrogens in the Aquatic Environment: A Review of Recent Risk Assessment Data.” AAPS Journal 16: 299–310. 24470211 10.1208/s12248-014-9561-3 PMC3933577

[emi470230-bib-0041] Le, T. C. , E. J. Lee , J. Lee , et al. 2019. “Saccharoquinoline, a Cytotoxic Alkaloidal Meroterpenoid From Marine‐Derived Bacterium *Saccharomonospora* sp .” Marine Drugs 17: 98. 30717397 10.3390/md17020098 PMC6410326

[emi470230-bib-0088] Lindqvist, L. L. , S. A. Jarmusch , E. C. Sonnenschein , et al. 2023. “Tropodithietic Acid, a Multifunctional Antimicrobial, Facilitates Adaption and Colonization of the Producer.” Phaeobacter piscinae. mSphere 8. 10.1128/msphere.00517-22. PMC9942592 36622251

[emi470230-bib-0042] Liu, J. J. , B. Pemberton , J. Lewis , P. J. Scales , and G. J. O. Martin . 2020. “Wastewater Treatment Using Filamentous Algae—A Review.” Bioresource Technology 298: 122556. 31843358 10.1016/j.biortech.2019.122556

[emi470230-bib-0043] Love, M. I. , W. Huber , and S. Anders . 2014. “Moderated Estimation of Fold Change and Dispersion for Rna‐Seq Data With Deseq2.” Genome Biology 15: 550. 25516281 10.1186/s13059-014-0550-8 PMC4302049

[emi470230-bib-0044] Løvlie, A. 1964. “Genetic Control of Division Rate and Morphogenesis in *Ulva mutabilis * Føyn.” Comptes‐Rendus des Travaux du Laboratoire Carlsberg 34: 77–168. 14153927

[emi470230-bib-0045] Ludmila, M. , L. Veronika , M. Alena , M. Tatyana , Z. Svetlana , and E. Victor . 2023. “Morphological Changes and Biochemical Reaction of *Ulva rigida* in Response to the Toxic Effect of Bisphenol a Under Experimental Conditions.” Aquatic Botany 184: 103579.

[emi470230-bib-0046] Luo, Y. L. , W. S. Guo , H. H. Ngo , et al. 2014. “A Review on the Occurrence of Micropollutants in the Aquatic Environment and Their Fate and Removal During Wastewater Treatment.” Science of the Total Environment 473: 619–641. 24394371 10.1016/j.scitotenv.2013.12.065

[emi470230-bib-0047] Martin, M. 2011. “Cutadapt Removes Adapter Sequences From High‐Throughput Sequencing Reads.” EMBnet Journal 17, no. 1: 10–12.

[emi470230-bib-0048] McMurdie, P. J. , and S. Holmes . 2013. “Phyloseq: An R Package for Reproducible Interactive Analysis and Graphics of Microbiome Census Data.” PLoS One 8: e61217. 23630581 10.1371/journal.pone.0061217 PMC3632530

[emi470230-bib-0049] Michalowicz, J. 2014. “Bisphenol A—Sources, Toxicity and Biotransformation.” Environmental Toxicology and Pharmacology 37: 738–758. 24632011 10.1016/j.etap.2014.02.003

[emi470230-bib-0050] Mohsenpour, S. F. , S. Hennige , N. Willoughby , A. Adeloye , and T. Gutierrez . 2021. “Integrating Micro‐Algae Into Wastewater Treatment: A Review.” Science of the Total Environment 752: 142168. 33207512 10.1016/j.scitotenv.2020.142168

[emi470230-bib-0051] Montiel‐Leon, J. M. , G. Munoz , S. V. Duy , et al. 2019. “Widespread Occurrence and Spatial Distribution of Glyphosate, Atrazine, and Neonicotinoids Pesticides in the St. Lawrence and Tributary Rivers.” Environmental Pollution 250: 29–39. 30981933 10.1016/j.envpol.2019.03.125

[emi470230-bib-0052] Mudlaff, C. M. , F. Weinberger , L. Düsedau , M. Ghotbi , S. Künzel , and G. Bonthond . 2025. “Seasonal Cycles in a Seaweed Holobiont: A Multiyear Time Series Reveals Repetitive Microbial Shifts and Core Taxa.” Environmental Microbiology 27: e70062. 40015318 10.1111/1462-2920.70062 PMC11867712

[emi470230-bib-0053] Nahor, O. , C. F. Morales‐Reyes , G. Califano , T. Wichard , A. Golberg , and Á. Israel . 2021. “Flow Cytometric Measurements as a Proxy for Sporulation Intensity in the Cultured Macroalga *Ulva* (Chlorophyta).” Botanica Marina 64: 83–92.

[emi470230-bib-0054] Nguyen, D. , O. Ovadia , and L. Guttman . 2023. “Temporal Force Governs the Microbial Assembly Associated With *Ulva* *fasciata* (Chlorophyta) From an Integrated Multi‐Trophic Aquaculture System.” Frontiers in Microbiology 14: 1223204. 37869666 10.3389/fmicb.2023.1223204 PMC10585273

[emi470230-bib-0055] Nguyen, L. M. , N. T. T. Nguyen , T. T. T. Nguyen , T. T. Nguyen , D. T. C. Nguyen , and T. V. Tran . 2022. “Occurrence, Toxicity and Adsorptive Removal of the Chloramphenicol Antibiotic in Water: A Review.” Environmental Chemistry Letters 20: 1929–1963. 35369683 10.1007/s10311-022-01416-x PMC8956153

[emi470230-bib-0056] Oksanen, J. 2022. Vegan: Community Ecology Package. Github.

[emi470230-bib-0057] Parada, A. E. , D. M. Needham , and J. A. Fuhrman . 2016. “Every Base Matters: Assessing Small Subunit rRNA Primers for Marine Microbiomes With Mock Communities, Time Series and Global Field Samples.” Environmental Microbiology 18: 1403–1414. 26271760 10.1111/1462-2920.13023

[emi470230-bib-0058] Qiu, S. , S. J. Ge , P. Champagne , and R. M. Robertson . 2017. “Potential of *Ulva* *lactuca* for Municipal Wastewater Bioremediation and Fly Food.” Desalination and Water Treatment 91: 23–30.

[emi470230-bib-0085] Qui‐Minet, Z. N. , T. Wichard , G. Del Olmo , et al. 2025. “Light‐Regulated Interactions between Phaeobacter sp. and Ulva ohnoi (Chlorophyta): Effects on Microbiome Dynamics, Metabolome Composition, and Tropodithietic Acid Production.” Environmental and Experimental Botany 230: 106093. 10.1016/j.envexpbot.2025.106093.

[emi470230-bib-0059] Rahhou, A. , M. Layachi , M. Akodad , et al. 2023. “The Bioremediation Potential of *Ulva lactuca* (Chlorophyta) Causing Green Tide in Marchica Lagoon (Ne Morocco, Mediterranean Sea): Biomass, Heavy Metals, and Health Risk Assessment.” Water 15: 1310.

[emi470230-bib-0060] Rose, M. T. , T. R. Cavagnaro , C. A. Scanlan , et al. 2016. “Impact of Herbicides on Soil Biology and Function.” Advances in Agronomy 136: 133–220.

[emi470230-bib-0061] Roth‐Schulze, A. J. , J. Pintado , E. Zozaya‐Valdes , et al. 2018. “Functional Biogeography and Host Specificity of Bacterial Communities Associated With the Marine Green Alga *Ulva* spp .” Molecular Ecology 27: 1952–1965. 29420863 10.1111/mec.14529

[emi470230-bib-0062] Saha, M. , S. M. Dittami , C. X. Chan , et al. 2024. “Progress and Future Directions for Seaweed Holobiont Research.” New Phytologist 244: 364–376. 39137959 10.1111/nph.20018

[emi470230-bib-0063] Seyedsayamdost, M. R. , R. J. Case , R. Kolter , and J. Clardy . 2011. “The Jekyll‐and‐Hyde Chemistry of *Phaeobacter gallaeciensis* .” Nature Chemistry 3: 331–335. 10.1038/nchem.1002 PMC3376411 21430694

[emi470230-bib-0064] Sousa, J. C. G. , A. R. Ribeiro , M. O. Barbosa , M. F. R. Pereira , and A. M. T. Silva . 2018. “A Review on Environmental Monitoring of Water Organic Pollutants Identified by Eu Guidelines.” Journal of Hazardous Materials 344: 146–162. 29032095 10.1016/j.jhazmat.2017.09.058

[emi470230-bib-0065] Spoerner, M. , T. Wichard , T. Bachhuber , J. Stratmann , and W. Oertel . 2012. “Growth and Thallus Morphogenesis of *Ulva* *mutabilis* (Chlorophyta) Depends on a Combination of Two Bacterial Species Excreting Regulatory Factors.” Journal of Phycology 48: 1433–1447. 27009994 10.1111/j.1529-8817.2012.01231.x

[emi470230-bib-0066] Steinhagen, S. , A. Barco , T. Wichard , and F. Weinberger . 2019. “Conspecificity of the Model Organism *Ulva* *mutabilis* and *Ulva* *compressa* (Ulvophyceae, Chlorophyta).” Journal of Phycology 55: 25–36. 30367499 10.1111/jpy.12804

[emi470230-bib-0067] Steinhilber, D. , M. Schubert‐Zsilavecz , and H. Roth . 2010. Medizinische Chemie. Deutscher Apotheker Verlag.

[emi470230-bib-0068] Stratmann, J. , G. Paputsoglu , and W. Oertel . 1996. “Differentiation of *Ulva* *mutabilis* (Chlorophyta) Gametangia and Gamete Release Are Controlled by Extracellular Inhibitors.” Journal of Phycology 32: 1009–1021.

[emi470230-bib-0069] Sun, J. , L. Steindler , J. C. Thrash , et al. 2011. “One Carbon Metabolism in Sar11 Pelagic Marine Bacteria.” PLoS One 6: e23973. 21886845 10.1371/journal.pone.0023973 PMC3160333

[emi470230-bib-0070] Tran, N. H. , M. Reinhard , and K. Y. Gin . 2018. “Occurrence and Fate of Emerging Contaminants in Municipal Wastewater Treatment Plants From Different Geographical Regions–A Review.” Water Research 133: 182–207. 29407700 10.1016/j.watres.2017.12.029

[emi470230-bib-0071] Trinelli, M. A. , M. M. Areco , and M. dos Santos Afonso . 2013. “Co‐Biosorption of Copper and Glyphosate by *Ulva lactuca* .” Colloids and Surfaces. B, Biointerfaces 105: 251–258. 23376752 10.1016/j.colsurfb.2012.12.047

[emi470230-bib-0072] Ulrich, J. F. , M. S. Gräfe , S. Dhiman , P. Wienecke , H.‐D. Arndt , and T. Wichard . 2022. “Thallusin Quantification in Marine Bacteria and Algae Cultures.” Marine Drugs 20: 690. 36355014 10.3390/md20110690 PMC9696546

[emi470230-bib-0073] van der Loos, L. M. , C. De Wilde , A. Willems , O. De Clerck , and S. Steinhagen . 2024. “The Cultivated Sea Lettuce (*Ulva*) Microbiome: Successional and Seasonal Dynamics.” Aquaculture 585: 740692.

[emi470230-bib-0074] van der Loos, L. M. , S. D'Hondt , A. H. Engelen , et al. 2023. “Salinity and Host Drive *Ulva*‐Associated Bacterial Communities Across the Atlantic‐Baltic Sea Gradient.” Molecular Ecology 32: 6260–6277. 35395701 10.1111/mec.16462

[emi470230-bib-0075] Viegas, C. , L. Gouveia , and M. Goncalves . 2021. “Aquaculture Wastewater Treatment Through Microalgal. Biomass Potential Applications on Animal Feed, Agriculture, and Energy.” Journal of Environmental Management 286: 112187. 33609932 10.1016/j.jenvman.2021.112187

[emi470230-bib-0076] Wahl, M. , F. Goecke , A. Labes , S. Dobretsov , and F. Weinberger . 2012. “The Second Skin: Ecological Role of Epibiotic Biofilms on Marine Organisms.” Frontiers in Microbiology 3: 292. 22936927 10.3389/fmicb.2012.00292 PMC3425911

[emi470230-bib-0077] Weiss, A. , R. Costa , and T. Wichard . 2017. “Morphogenesis of *Ulva* *mutabilis* (Chlorophyta) Induced by *Maribacter* Species (Bacteroidetes, Flavobacteriaceae).” Botanica Marina 60: 197–206.

[emi470230-bib-0078] Wichard, T. 2015. “Exploring Bacteria‐Induced Growth and Morphogenesis in the Green Macroalga Order Ulvales (Chlorophyta).” Frontiers in Plant Science 6: 86. 25784916 10.3389/fpls.2015.00086 PMC4347444

[emi470230-bib-0079] Wichard, T. 2023. “From Model Organism to Application: Bacteria‐Induced Growth and Development of the Green Seaweed *Ulva* and the Potential of Microbe Leveraging in Algal Aquaculture.” Seminars in Cell and Developmental Biology 134: 69–78. 35459546 10.1016/j.semcdb.2022.04.007

[emi470230-bib-0080] Zilber‐Rosenberg, I. , and E. Rosenberg . 2008. “Role of Microorganisms in the Evolution of Animals and Plants: The Hologenome Theory of Evolution.” FEMS Microbiology Reviews 32: 723–735. 18549407 10.1111/j.1574-6976.2008.00123.x

