# Supplementary material for: The Microbiome of the Seaweed Cultivar Ulva compressa (Chlorophyta) and Its Persistence Under Micropollutant Exposure
Source: Environ Microbiol Rep. 2025 Nov 10;17(6):e70230. doi: 10.1111/1758-2229.70230 (PMC12602998; doi:10.1111/1758-2229.70230)
Supplement: Supplementary file 1 — Table S1: Experiment parameters for the addition of chemical stressors. Table S2: Genbank accession numbers. Samples as part of the BioProject PRJNA828511 (NCBI). Table S3: Overview of the determined amplicon sequence variances in various treatments. Number of replicates reads (average of replicates), maximum observed ASVs and alpha diversity (average of replicates) for lab cultures, treated samples and five environmental samples. Figure S1: Rarefraction curves of bacterial richness, Shannon and Simpson. Replicates within each treatment displayed consistent profiles, indicating good reproducibility of the sequencing data. Control samples exhibited higher or similar diversity as pollutant‐exposed samples. Antibiotic treatments, particularly erythromycin (ERY), chloramphenicol (CAP) and oxytetracycline (OTC), caused the most substantial reductions. Treatments with endocrine disruptors (BPA, E2 and EE2) showed intermediate effects on microbial richness and diversity. In contrast, environmental samples (Ria Formosa: RFM #4–8) displayed substantially higher richness compared to long‐term cultured Ulva, highlighting the overall reduction of microbiome diversity under laboratory cultivation. [file EMI4-17-e70230-s001.docx]

**Supporting Material for**

**The microbiome of the seaweed cultivar *Ulva compressa* (Chlorophyta) and its persistence under micropollutant exposure**

Justus Hardegen^1^, Gabriel Amend^1^, Thomas Wichard^1,2,*^

^1^ Friedrich Schiller University Jena, Institute for Inorganic and Analytical Chemistry, Lessingstr. 8, 07743 Jena, Germany

^2^ Jena School for Microbial Communication, 07743 Jena, Germany

^*^ Corresponding Author: thomas.wichard@uni-jena.de

Contents

[Table S1: Experiment parameters for the addition of chemical stressors 2](#_Toc212463484)

[Table S2: Genbank accession numbers 3](#_Toc212463485)

[Table S3: Overview of the determined amplicon sequence variances in various treatments 4](#_Toc212463486)

[Table S4: Summary of the phenotypes of the identified bacterial species and the ASV sequences used for the microbiome analysis. 5](#_Toc212463487)

[Figure S1: Rarefraction curves of bacterial richness, Shannon and Simpson 6](#_Toc212463488)

# Table S1: Experiment parameters for the addition of chemical stressors

| Types of stressors | Treatment | Abbreviation | Concentration (mg L^−1^) | Final methanol content (*v*/*v*) | Number of replicates |
| --- | --- | --- | --- | --- | --- |
| Antibiotics | Chloramphenicol | CAP | 262 | 2.1 % | 4 |
|  | Erythromycin | ERY | 270 | 2.1 % | 4 |
|  | Oxytetracycline | OTC | 54.3 | 2.1 % | 4 |
|  | Sulfamethoxazole | SMX | 201 | 2.1 % | 4 |
| Herbicides | Atrazine | ATZ | 3.26 | 0.07 % | 4 |
|  | Glyphosate | PMG | 152 | 0 % | 4 |
| Endocrine | Bisphenol A | BPA | 13.2 | 0.07 % | 4 |
| disruptors | Estradiol | E_2_ | 3.60 | 0.07 % | 4 |
|  | Ethinylestradiol | EE_2_ | 2.83 | 0.06 % | 4 |
| Controls | Methanol | MeOH | 0 | 2.1 % | 4 |
|  | Negative | Ctrl 0 d / 14 d | 0 | 0 % | 5/5 |

Table S2: Genbank accession numbers. Samples as part of the BioProject PRJNA828511 (NCBI).

| Treatment | Accesion  numbers | Strain/  Isolate |
| --- | --- | --- |
| Ctrl 0 d | SAMN48728462 – SAMN48728466 | FSU-UM1-41 |
| Ctrl 14 d | SAMN48728467 – SAMN48728471 | FSU-UM1-41 |
| MeOH | SAMN48728484 – SAMN48728487 | FSU-UM1-41 |
| ATZ | SAMN48728450 – SAMN48728453 | FSU-UM1-41 |
| BPA | SAMN48728454 – SAMN48728457 | FSU-UM1-41 |
| CAP | SAMN48728458 – SAMN48728461 | FSU-UM1-41 |
| E2 | SAMN48728472 – SAMN48728475 | FSU-UM1-41 |
| EE2 | SAMN48728476 – SAMN48728479 | FSU-UM1-41 |
| ERY | SAMN48728480 – SAMN48728483 | FSU-UM1-41 |
| OTC | SAMN48728488 – SAMN48728491 | FSU-UM1-41 |
| PMG | SAMN48728492 – SAMN48728495 | FSU-UM1-41 |
| SMX | SAMN48728496 – SAMN48728499 | FSU-UM1-41 |
| Environmental | SAMN48728500 – SAMN48728504 | Isolate RFU-61 |

Table S3: Overview of the determined amplicon sequence variances in various treatments**.** Number of replicates reads (average of replicates), maximum observed ASVs, and alpha diversity (average of replicates) for lab cultures, treated samples, and five environmental samples.

| Condition | Number of replicates | Number of reads | Observed ASVs | Simpson diversity | Shannon diversity |
| --- | --- | --- | --- | --- | --- |
| Ctrl 0 d | 5 | 38131 | 49 | 0.0738 | 2.91 |
| Ctrl 14 d | 5 | 40649 | 48 | 0.0660 | 3.01 |
| MeOH | 4 | 65697 | 45 | 0.2533 | 2.23 |
| ATZ | 4 | 50747 | 48 | 0.0700 | 2.98 |
| BPA | 4 | 53892 | 46 | 0.1045 | 2.80 |
| CAP | 4 | 46194 | 44 | 0.2178 | 2.16 |
| E2 | 4 | 48742 | 49 | 0.0700 | 3.01 |
| EE2 | 4 | 50494 | 49 | 0.0671 | 3.03 |
| ERY | 4 | 40541 | 45 | 0.1317 | 2.54 |
| OTC | 4 | 76423 | 47 | 0.3525 | 1.91 |
| PMG | 4 | 59942 | 48 | 0.0644 | 2.99 |
| SMX | 4 | 52479 | 49 | 0.0615 | 3.06 |
| Environmental | 1 | 58370 | 146 | 0.2500 | 2.37 |
| Environmental | 1 | 45795 | 140 | 0.0345 | 3.84 |
| Environmental | 1 | 47865 | 144 | 0.0831 | 3.35 |
| Environmental | 1 | 18019 | 122 | 0.0789 | 3.33 |
| Environmental | 1 | 56953 | 157 | 0.0437 | 3.83 |

Table S4: Summary of the phenotypes of the identified bacterial species and the ASV sequences used for the microbiome analysis. See the accompanying Excel file *“Hardegen et al. Table S4 Selected ASV 180625.xlsx”* in the Supplementary Material for full details.

**Figure S1**


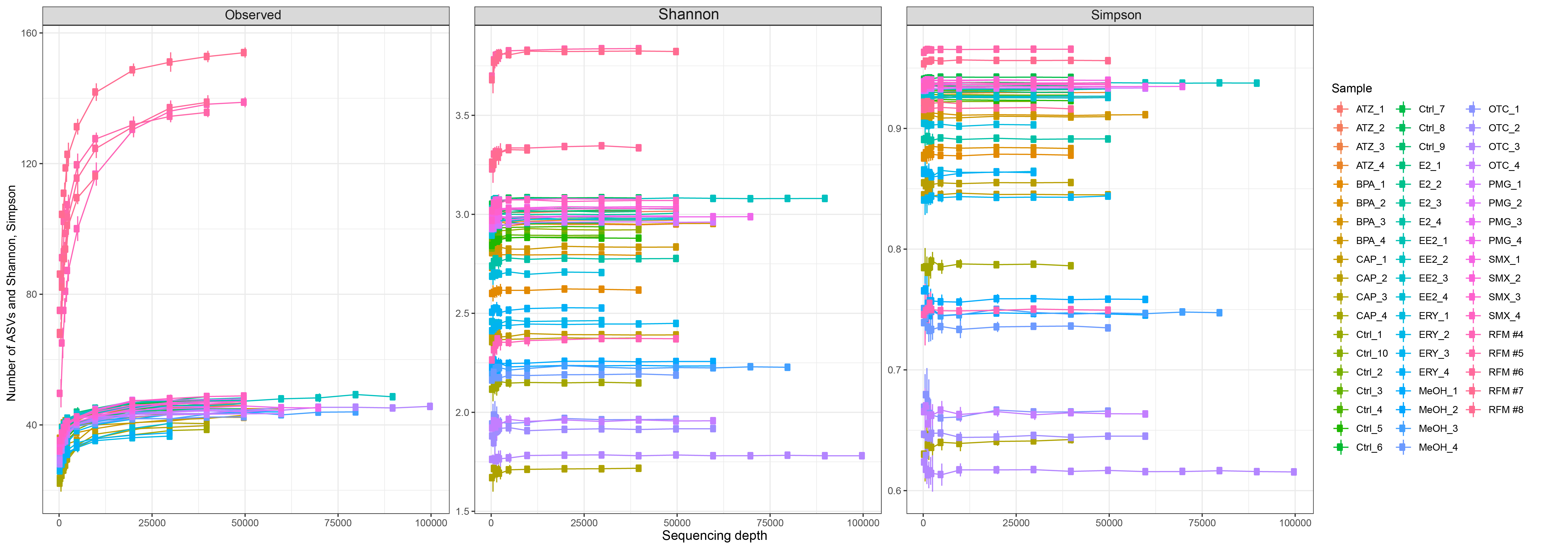


Figure S1: Rarefraction curves of bacterial richness, Shannon and Simpson. Replicates within each treatment displayed consistent profiles, indicating good reproducibility of the sequencing data. Control samples exhibited higher or similar diversity as pollutant-exposed samples. Antibiotic treatments, particularly erythromycin (ERY), chloramphenicol (CAP), and oxytetracycline (OTC), caused the most substantial reductions. Treatments with endocrine disruptors (BPA, E2, EE2) showed intermediate effects on microbial richness and diversity. In contrast, environmental samples (Ria Formosa: RFM #4-8) displayed substantially higher richness compared to long-term cultured *Ulva*, highlighting the overall reduction of microbiome diversity under laboratory cultivation.
